# The Evaluation Method of Low-Carbon Scenic Spots by Combining IBWM with B-DST and VIKOR in Fuzzy Environment

**DOI:** 10.3390/ijerph17010089

**Published:** 2019-12-21

**Authors:** Aijun Liu, Taoning Liu, Xiaohui Ji, Hui Lu, Feng Li

**Affiliations:** 1 Department of Management Engineering, School of Economics & Management, Xidian University, Xi’an 710071, China; ajliu@xidian.edu.cn (A.L.); tnliu@stu.xidian.edu.cn (T.L.); xhji@stu.xidian.edu.cn (X.J.); 2 Tianhua College, Shanghai Normal University, Shanghai 201815, China; janetluck@126.com; 3 School of Information, Beijing Wuzi University, Beijing 101149, China

**Keywords:** low-carbon scenic spots, multicriteria group decision making, IBWM, B-DST, VIKOR, low-carbon

## Abstract

With the concept of sustainability gaining popularity, low-carbon tourism has been widely considered. In this paper, a multicriteria group decision making (MCGDM) process based on an uncertain environment is proposed to study the evaluation problem of low-carbon scenic spots (LSSs). In order to minimize the influence of subjective and objective factors, the traditional Vlse Kriterjumska Optimizacija I Kompromisno Resenje (VIKOR) method is expanded, using the improved best and worst method (IBWM) and Bayes approximation method, based on Dempster-Shafer Theory (B-DST). First, in order to make the evaluation process more professional, a number of evaluation criteria are established as effective systems, followed by the use of triangular intuitionistic fuzzy numbers (TIFNs) to evaluate alternatives of LSSs. Next, according to the evaluation results, the weights of the criteria are determined by the IBWM method, and the weights of the expert panels (Eps) are determined by B-DST. Finally, a weighted averaging algorithm of TIFN is used to integrate the above results to expand the traditional VIKOR and obtain the optimal LSS. The applicability of this method is proven by example calculation. The main conclusions are as follows: tourist facilities and the eco-environment are the two most important factors influencing the choice of LSSs. Meanwhile, the roles of management and participant attitudes in LSS evaluations cannot be ignored.

## 1. Introduction

Low-carbon tourism was first proposed during the 2009 World Economic Forum “towards low-carbon tourism” [1]. As a means of sustainable development, low-carbon tourism can promote economic growth and social development. The study found that carbon dioxide emissions from tourism development account for 4.4% of total global carbon emissions. And by 2035, emissions are expected to grow at an average rate of 3.2% per year [2]. Therefore, the role of tourism in the development of a low-carbon economy cannot be ignored [3]. LSSs are an important carrier for low-carbon tourism. While on holidays, tourists generate a lot of carbon emissions, including through food, accommodation, traffic, visits, shopping, and entertainment. Therefore, more and more scenic spots are beginning to actively respond to the call for the implementation of low-carbon policies in the development process. Many tourist facilities in the Yanzigou Scenic Spot in Sichuan province are specially designed to meet low carbon demands. For example, the garbage bins in the scenic area are humanized, and there are slogans which remind visitors to make an effort to maintain environmental sanitation.

However, academic research on LSSs has lagged behind. Therefore, it is necessary to understand the connotation of LSS and establish an evaluation system as quickly as possible. This would provide not only a scientific basis for macro carbon emission reduction decisions, but also theoretical guidance for future LSS criteria verification, emission reduction project cooperation, and the establishment of an emission compensation system. At present, most published studies have focused on qualitative analyses, while few quantitative analyses have been published. Moreover, studies of scenic spots have only focused on the construction of a system of criteria [4,5,6].

**Figure 1:**
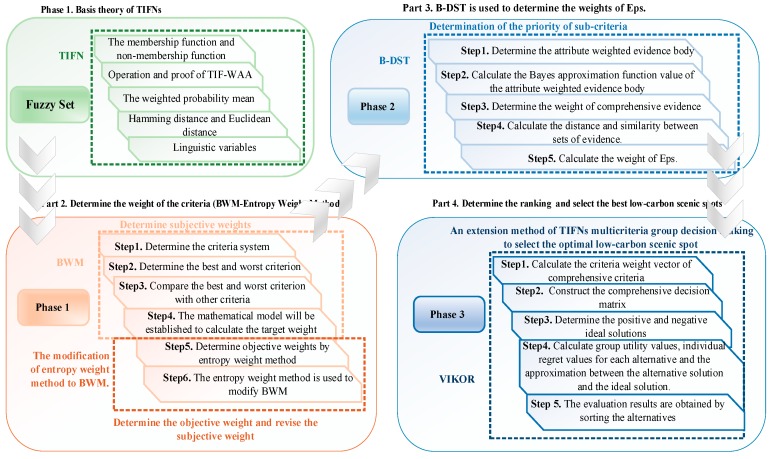
Steps of the evaluation method.

In this paper, the evaluation of LSSs is studied by combining TIFNs, IBWM, B-DST, and the expanded VIKOR method (as shown in Figure 1). The IBWM is used to determine the criteria weights of LSSs. BWM is a very effective multicriteria decision method (MCDM) which is used to determine criteria weights. It was proposed in 2015 as a new approach [7], so it has some shortcomings, which are mainly reflected in two aspects. On the one hand, in the process of using traditional BWM, the best and worst criteria are determined only by the subjective decisions of experts. On the other hand, the 1–9 scale is insufficient to express the difference between the best and worst criteria [8]. In this paper, an entropy weight method and TIFNs are used to make up for these shortcomings. The entropy weight method is introduced to modify the subjective weight. For the second deficiency, TIFNs is used as the evaluation language to measure the deviation degree of the difference criterion. At present, there are many fuzzy languages; TIFNs was chosen because it can better express the hesitation degree of decision-making problems in reality. In addition, due to the complexity of information in MCGDM, a single decision maker is often affected by subjective factors, and cannot represent the comprehensiveness of the problem. In this case, multiple experts from different fields are required to participate in the decision. Therefore, the weight information determination of decision makers is a particularly important research field. B-DST is an extension of Dempster-Shafer Theory (DST), which is different from DST in terms of knowing the prior probability. In addition, it is a good way to express the difference between “uncertain” and “don’t know”, and the reasoning form is not complicated. Therefore, it is widely used in uncertain environments [9,10]. The criteria and expert weights can be obtained effectively through the above two methods; then, VIKOR is selected to determine the final ranking of alternative LSSs. VIKOR is actually a compromise sorting method, by maximizing the group benefits and minimizing the individual regret to compromise a sort limited decision scheme in different evaluation standards and complicated decision environments. Furthermore, it can effectively avoid subjectivity and uncertainty problems, and has high levels of reliability and rationality [11,12]. Finally, some of the major findings are summarized as the following related management science point of view: (1) The development of low-carbon tourism is the inevitable trend of the sustainable development of scenic spots, which requires both managers and tourists to have a low-carbon thinking, to focus on the overall situation, and to actively participate in low-carbon construction, so as to make scenic spots develop in a sustainable manner. (2) The construction of LSS can be effectively combined with modern electronic information technology to promote the intelligent development of scenic spot tourism. Scenic spots can start from the following points: Firstly, the construction of digital scenic spots, the integration of scenic spots planning, scenic spots protection, scenic spots services, and other information. Secondly, electronic tour guides can be developed. Finally, scenic spots can use electronic tickets. This is not only conducive to low-carbon management; low-carbon publicity can also play a role in increasing the level of enthusiasm of tourists. (3) Conditional scenic spots can use regional or ethnic characteristics transport (such as horse-drawn cart, camel manned), and provide battery cars, bicycles, or feature low-carbon vehicles for the convenience of tourists.

The structure of this paper is as follows: Section 2 presents a literature review including low carbon tourism and the methods used in the evaluation process. Section 3 establishes a criteria system for evaluating LSSs. Section 4 presents the proposed integrated framework for LSS criteria evaluation. Section 5 presents an example on LSS criteria evaluation to validate the proposed model, and the different sorting results obtained by different parameters and methods are discussed. Section 6 concludes the research process and puts forward some constructive suggestions.

## 2. Literature Review

Since this paper contains two major innovations, the literature review is divided into two aspects: The first presents a status analysis of LSS research, while the second presents an analysis of the MCDM used for the evaluation system.

The study of low-carbon tourism was first introduced in Europe and the United States [13]. Since the 1990s, with the emerging energy crisis and environmental pollution [14], several new types of tourism development have emerged, such as green tourism and eco-tourism. The general population is also increasingly focused on the impact of tourism development on the environment [15], especially with regard to carbon emissions, which has also received a good deal of expert attention. With the rise of research on energy utilization and greenhouse gas emissions, research on low-carbon tourism has mainly focused on the relationship between tourism and global climate change, investigating their interactions, as well as low-carbon tourism services. For example, the researchers investigated and came up with a quantitative measure of carbon emissions from travel-related traffic [16,17]. Peeters and Dubois argued that international aviation and private cars are dominant factors in high-carbon tourism [2]. Kuo and Chen used the LCA evaluation method to quantitatively study energy use, greenhouse gas emissions, wastewater, and solid waste related to tourism [18]. They concluded that both tourist consumption and waste emissions exceeded the daily usage of local residents. Becken et al. reported that the energy consumption of tourism has a strong correlation with the behavior of tourists [19]. Lin reported that the carbon emissions of private cars are more harmful to the environment than those of other traffic tools [20]. Tol suggested that a carbon tax affects the choice of tourists regarding tourism destinations [21]. Therefore, its implementation can reduce emissions to some extent. Similarly, current international research on low-carbon tourism services is focused on resource conservation and the environmental friendliness of tourism services. Various tourism service models have been proposed that reflect the low-carbon concept, and which are based on the protection of the ecological environment [22]. The practice model of low-carbon tourism services is constantly being enriched, e.g. by Spain’s green energy-saving tourism, Germany’s comprehensive tourism, Japan’s environmental preservation tourism, Korea’s environmentally-friendly tourism, and Israel’s water-saving rural tourism. In addition, research on the evaluation methods of tourism service provision efficiency is becoming more substantive. Methods such as random value evaluation, the ecological effect method, the fuzzy comprehensive evaluation method, the ecological footprint method, and the multisector dynamic macroeconomic model have been used widely. For example, Blancas et al. used random value evaluation to analyze the efficiency of the utilization of resources in Spanish ecotourism [23]. Michalena et al. used the general equilibrium and multisector dynamic macroeconomic models to analyze the efficiency of the tourism industry [24].

Few relevant studies on low-carbon tourism have been published. The LSS is a source of low-carbon tourism, and also a key link in carbon emissions in tourism, which is of great significance for low-carbon tourism. In addition, the existing literature on scenic spots has mainly focused on evaluation criteria [25]. Low-carbon tourism scenic spots cannot be simply equated with energy conservation and emissions reductions, but should be extended to the four stages of resources and the environment, emission reduction technology, consumption management, and policy philosophy. Qian et al. divided the evaluation criteria for LSSs into four aspects which are relevant for the eco-environment, tourist facilities, management system, and participant attitudes using Delphi, and further expanded these aspects into two levels, thereby obtaining 27 four-level criteria [4]. The Xixi wetland was used as an example to verify the validity of the research. Among the existing studies, no evaluation method has been proposed for low-carbon tourist attractions. Therefore, this study proposes a MCDM method for the evaluation of scenic spots which converts the demands of tourists regarding scenic spots into the criteria of a LSS construction. Moreover, the relationship between the most important scenic spot criteria was also considered, which plays an important role in the construction of new scenic spots.

After a long period of development, the MCDM method has made some achievements. At present, common methods include the Analytic Hierarchy process (AHP), Network Analytic Hierarchy Process (ANP), Decision Experiment and Evaluation Experiment (DEMATEL), Technique for Order Preference by Similarity to an Ideal Solution (TOPSIS), Linear Programming (LP), and the Data Envelope-Analysis method (DEA). In 1998, Opricovic proposed a VIKOR method which is applicable to MCDM technology, emphasizing the selection and ordering of alternative sets of conflict criteria [26,27]. It represents the distance from the positive ideal solution, taking into account the relative importance of all criteria, and the balance between overall satisfaction and individual satisfaction [28]. In order to effectively solve the disadvantage of fuzzy uncertainty in MCDM, Tian introduced triangular fuzzy number (TFN) theory into the MCDM method for the evaluation of a smart bike-sharing program (BSP), and language variables were used to quantify the performance of the alternatives [29]. Li and Zhao proposed a fuzzy GRA-VIKOR assessment of an ecological industrial power plant, and combined fuzzy AHP with a fuzzy entropy weight method to obtain subjective and objective comprehensive weights [30], which could be used to deal with the uncertainty in the decision-making process. Therefore, this paper applies the TIFN theory to effectively quantify fuzzy, subjective, objective, and uncertain linguistic variables. When using the MCDM method to evaluate LSSs, in addition to effectively quantifying the language variables, another issue that needs attention is determining the importance of different criteria and different Eps, so appropriate methods should be taken to determine the weight [31]. Wan used the entropy weight method to calculate the weight values of many supplier attributes [32]. Xu combined the subjective weight obtained by the G1 method with the objective weight obtained by the entropy method to determine the comprehensive weight of the attributes [33]. As a very effective multiattribute decision making method to gain the subjective weight, BWM was proposed by Rezaei in 2015, and was extended in 2016 [34]. In this paper, through the BWM method, the subjective weights of the LSS evaluation criteria are obtained, and the entropy weight method is used to modify them, which will make the evaluation results more accurate. At the same time, due to the complexity of decision information, it is often difficult for a single decision-maker to operate alone; Rather Eps from different fields are required to make decisions together. Therefore, the determination of weight information of decision-makers has become a particularly important research field. DST was formally put forward by Harvard University mathematician A.P.Dempster [35]. Subsequently, in 1976, his student Shafer further improved the theory [36]. Tang defined an aggregation operator within the framework of intuitionistic fuzzy multicriteria [37]. Liu and Gao combined the intuitionistic fuzzy set (IFS) with the DST to obtain expert weights in decision making [38]. The main content of this study is to solve the evaluation problems related to LSSs through a new MCDM method. In addition, this paper pays more attention to the establishment for a LSS criteria system in the evaluation process, which makes the decision-making results more comprehensive and scientific.

## 3. LSSs Evaluation Criteria System

**Figure 2:**
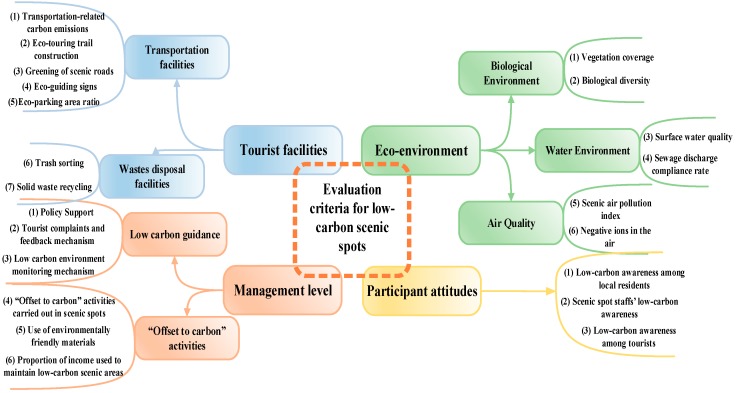
Evaluation criteria for LSSs.

In order to comprehensively evaluate LSSs, a complete evaluation criteria system should be established. Therefore, the realization of LSS was mainly carried out from four aspects: eco-environment, tourism facilities, management system, and participant attitudes. The specific criteria system is shown in Figure 2.

### 3.1. Eco-Environment

LSS is composed of natural resource-type scenic spots, such as wetlands and forests. There are three main aspects to the evaluation for scenic resources: biological environment, water environment and air quality. The unique low-carbon landscape in the scenic area is the premise for the development of low-carbon tourism, and it is also the key to attracting tourists [39]. The type and quantity of vegetation affect the ecological environment of the scenic spot. The water environment, air quality, and the environment around the scenic spot are important evaluation criteria for the eco-environment in the scenic spot [40,41].

(1) Refers to the sum of all plant communities in a certain area. This criterion is usually used to reflect the green status in the scenic area.

(2) The quality of the ecological environment in the scenic spot is mainly reflected in the maintenance of species diversity in the scenic area.

(3) Refers to the surface water environmental quality standard of the People’s Republic of China (GB3838-2002); surface waters includes rivers, lakes, canals, channels, reservoirs, and so on.

(4) The drainage of scenic spots must meet the national standard (GB8978-88) comprehensive sewage discharge standard.

(5) Simplifies the concentration of several air pollutants that are routinely monitored into a single conceptual index value form.

(6) The air negative ion concentration is used as the basic observation index, and the air quality is evaluated by the unipolar and the air ion evaluation coefficients

### 3.2. Tourist Facilities

To build a LSS, the facilities inside the scenic spot should have low-carbon and environmental protection effects, mainly including two aspects: transportation facilities and waste disposal facilities. These two points play an important role in the construction for LSSs, and are also important criteria for evaluating the carbon level in the area [42]. In the process of transportation, huge carbon emissions will be generated [43].

(1) Tourist attractions mainly choose green transportation modes such as battery cars and mountain bikes. At the same time, motor vehicles are prohibited from entering, reducing pollution and reducing carbon emissions from scenic traffic.

(2) Road construction in the scenic area should select materials that are in harmony with the natural environment, reduce the proportion of hardened roads, and set up animal passages and reminder signs.

(3) The road construction for scenic spots needs to increase the greening rate.

(4) For the guidance information in the scenic area, original materials such as stones, wood, and pebbles can be selected.

(5) The scenic ecological parking lot generally uses shrub as the isolation line and should minimize the area of the hardened parking lot.

(6) Set up sorting bins in the scenic spot, and recycle and treat the recyclable garbage and non-recyclable garbage separately.

(7) The disposal of solid waste should be treated in such a way that it minimizes the impact on the environment.

### 3.3. Management Level

Management level is a key factor by which to determine the quality of LSSs. Generally, management levels are divided into hard and soft management. Hard management mainly refers to some hard criteria such as management target compliance rates, policy support, implementation intensity, travel complaints and feedback mechanisms, low-carbon operating systems, and supervision agencies [44]. Software management mainly has two aspects: low-carbon publicity education and low-carbon tourism penetration. Although the soft management method in this area is not as strict as the hard target requirements, the role is equally important. Through the slowly infiltrating method of making brochures, signboards, etc., the low-carbon concept is now deeply rooted in the hearts of the people [45].

(1) National policy support will help to stimulate scenic spots in accelerating the promotion of low-carbon tourism and forming a cyclical model of the entire industrial chain.

(2) Reference to the people-oriented concept, i.e., one which is in line with the development needs of LSSs, and, at the same time, which is better able to protect the interests of travelers.

(3) The water and air quality of scenic spots are vital, and the corresponding detection mechanisms must be established to sensitively detect environmental changes.

(4) Install a device that calculates the carbon emissions of tourists. Furthermore, and a tree field should be set up for visitors. By purchasing planting seedlings, the “offset to carbon activities” of tourism can be achieved.

(5) Tourism products in the scenic spot can be more localized agricultural products, and tourism packaging is as ecological as possible, thereby avoiding the waste of resources caused by excessive packaging.

(6) In order to maintain the ecological environment and maintain the low-carbon effect, the scenic spot must invest a certain amount of its resources every year into maintenance.

### 3.4. Participant Attitudes

The participants in low-carbon tourism include tourists and local residents. Tourists are the ultimate consumers of low-carbon tourism products [46]. While visiting scenic spots, tourists’ understanding of the low-carbon tourism concept will have an impact on their behavior regarding six aspects: food, accommodation, traffic, travel, visits, and entertainment. At present, low-carbon tourism is being recognized by more and more tourists. Local residents enjoy a good ecological and living environment brought about by low-carbon tourism resources. Their daily production and lifestyle should also meet a low-carbon standard [41]. Otherwise, it may indirectly have a destructive impact on LSSs.

(1) The attitude and participation of local residents toward low-carbon and environmental protection have an impact on the low-carbon operation of the scenic spot.

(2) Through the dissemination of low-carbon knowledge, all employees in the scenic area will influence tourists regarding their own low-carbon environmental behavior.

(3) Advocate low-carbon tourism and green tourism. Publicize and introduce low-carbon knowledge in public information materials.

## 4. Methodology

A novel MCDM method is introduced in this paper which combines TIFN, extended BWM, B-DST, and the extended VIKOR approach. Among these, TIFN is the basic evaluation language; extended BWM and combining evidence theory with Bayes approximation are mainly used to calculate the criteria and group decision makers, respectively. Extended VIKOR is the main research method.

### 4.1. Fuzzy Set Theory

**Figure 3:**
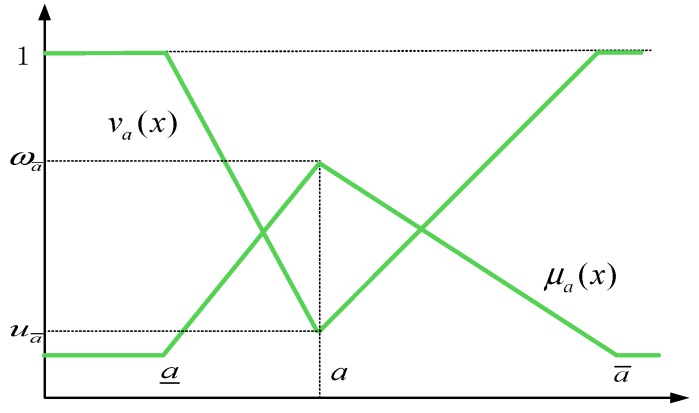
Functional distribution of TIFNs.

The fuzzy set theory proposed by Zadeh aims to simulate the ambiguity or inaccuracy of human cognitive processes [47]. A fuzzy number is a particular fuzzy set: $\tilde{a}=\left\{(x.\mu_{\tilde{a}}(x)).x\in R\right\}$ and μ(x) are a consecutive mapping from R to the closed interval [0,1]. TIFN is an extension of the discourse space of an intuitionistic fuzzy number (IFN) from a discrete set to continuous set. Compared with the definition of IFS, TIFN relates the membership and non-membership degrees to the fuzzy concept of “excellent’’ or ‘‘good’’ by adding triangular fuzzy number (TFN) (a, b, c), so that the decision information of different dimensions can be expressed more accurately. It is possible to use TIFNs to process data under uncertain conditions. The functional distribution of TIFNs is shown in Figure 3.

**Definition** **1.** *Let $\tilde{a}=\left(\left(\underline{a},a,\bar{a}\right);\omega_{\bar{a}},u_{\bar{a}}\right)$ be a TIFNs on real number set R; its membership function and non-membership function can be represented as follows:*

(1) $$\mu_{\tilde{a}}(x)=\{\begin{array}{cc} 0, & x<\underline{a}\\ \frac{x-\underline{a}}{a-\underline{a}}\omega_{\bar{a}}, & \underline{a}\leq x<a\\ \omega_{\bar{a}}, & x=a\\ \frac{\bar{a}-x}{\bar{a}-a}\omega_{\bar{a}}, & a<x\leq \underline{a}\\ 0 & x\geq \underline{a} \end{array}$$

(2) $$v_{\tilde{a}}(x)=\{\begin{array}{cc} \frac{a-x+(x-\underline{a})}{a-\underline{a}}u_{\bar{a}}, & \underline{a}\leq x<a\\ u_{\bar{a}}, & x=a\\ \frac{x-a+(\bar{a}-x)}{\bar{a}-a}u_{\bar{a}}, & a\leq x\leq \bar{a}\\ 1, & x<\underline{a}\;or\;x>\bar{a} \end{array}$$

*As shown in Figure 3, where*$\omega_{\bar{a}}$*and*$u_{\bar{a}}$*respectively represent the maximum membership degree and the minimum non-membership degree, such that they satisfy the condition:*$0\leq \omega_{\bar{a}}\leq 1$*,*$0\leq u_{\bar{a}}\leq 1$*and*$\omega_{\bar{a}}+u_{\bar{a}}\leq 1$.

**Definition** **2.** *The basic operation process of TIFN is described by Wan in detail [31].*

**Definition** **3.** *For the TIFN* ${\tilde{a}}_{r}=\left(\left({\underline{a}}_{r},a_{r},{\bar{a}}_{r}\right);\omega_{r},u_{r}\right)\;\left(r=1,2,...,k\right)$ *, the weighted averaging algorithm (WAA) is defined as Equation (3) (The proof of Equation (3) is provided in Appendix A.1).*

(3) $$TI-WAA\left({\tilde{a}}_{1},{\tilde{a}}_{2},...,{\tilde{a}}_{k}\right)=\sum_{r=1}^{k}w_{r}{\tilde{a}}_{r}=\left(\left(\sum_{r=1}^{k}w_{r}{\underline{a}}_{r},\sum_{r=1}^{k}w_{r}a_{r},\sum_{r=1}^{k}w_{r}{\bar{a}}_{r}\right);\underset{r}{\min}\omega_{\tilde{a}r},\underset{r}{\max}u_{\tilde{a}r}\right)$$

**Definition** **4.** *If* ${\tilde{a}}_{r}=\left(\left({\underline{a}}_{r},a_{r},{\bar{a}}_{r}\right);\omega_{r},u_{r}\right)\;\left(r=1,2,...,k\right)$ *is a set of TIFN. The weighted probability mean* $m^{d}$ *is calculated as follows:*

(4) $$m^{d}=\frac{1}{12}\left({\underline{a}}_{r}+4a_{r}+{\bar{a}}_{r}\right)\left[\left(1-u_{r}\right)+\omega_{r}\right]$$

**Definition** **5.** *The similarity measurement between* ${\tilde{a}}_{1}=\left(\left({\underline{a}}_{1},a_{1},{\bar{a}}_{1}\right);\omega_{1},u_{1}\right)$ *and* ${\tilde{a}}_{2}=\left(\left({\underline{a}}_{2},a_{2},{\bar{a}}_{2}\right);\omega_{2},u_{2}\right)$ *is calculated using the normalized Hamming distance and Euclidean distance, which are used to measure the shortest distance between two fuzzy numbers, as shown in Equations (5) and (6).*

(5) $$d_{h}\left({\tilde{a}}_{1},{\tilde{a}}_{2}\right)=\frac{1}{3}\left(\left|{\underline{a}}_{1}-{\underline{a}}_{2}\right|+\left|a_{1}-a_{2}\right|+\left|{\bar{a}}_{1}-{\bar{a}}_{2}\right|+\max\left(\left|\omega_{1}-\omega_{2}\right|,\left|u_{1}-u_{2}\right|\right)\right)$$

(6) de(a˜1,a˜2)=13{((a_1−a_2)2+(a1−a2)2+(a¯1−a¯2)2)+max(|ω1−ω2|2,|u1−u2|2)}12

*The above two distance of TIFNs formulas have the following properties:*

*(1)* $d\left({\tilde{a}}_{1},{\tilde{a}}_{2}\right)\geq 0$
*(2)* $d\left({\tilde{a}}_{1},{\tilde{a}}_{2}\right)=d\left({\tilde{a}}_{2},{\tilde{a}}_{1}\right)$
*(3) If* $\tilde{b}=\left(\left(\underline{b},b,\bar{b}\right);\omega_{\tilde{b}},u_{\tilde{b}}\right)$ *is any TIFN, then* $d\left({\tilde{a}}_{1},\tilde{b}\right)\leq d\left({\tilde{a}}_{1},\tilde{b}\right)+d\left({\tilde{a}}_{2},\tilde{b}\right)$

*(The proof of property (3) is provided in Appendix A.2)*

**Figure 4:**
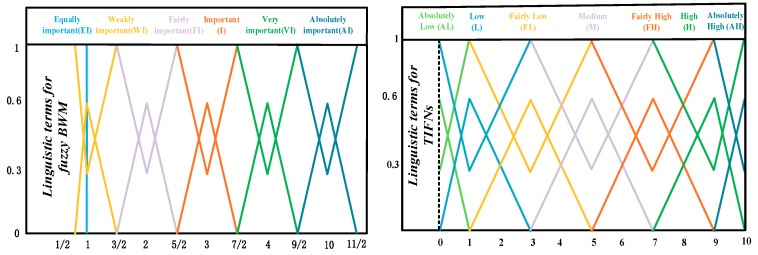
Linguistic terms.

**Definition** **6.** *In TIFN, language terms can be effectively converted into TIFNs by transforming scales. Table 1 lists the fuzzy BWM linguistic variables and consistency indices (CIs). Table 2 lists the linguistic variables for experts, rating the relationship between alternatives and criteria. Figure 4 shows the linguistic variables more intuitively on the axis.*

### 4.2. The Proposed Framework

Suppose that there are p Eps $\left\{DM_{1},DM_{2},...,DM_{p}\right\}$ to evaluate m alternatives $\left\{B_{1},B_{2},...,B_{m}\right\}$; each alternative is composed of n criteria values $CR_{j}(j=1,2,...,n)$. Let $W_{CR}^{k}={\left(w_{CR,1}^{k},w_{CR,2}^{k},...,w_{CR,n}^{k}\right)}^{T}$ be the weight vector, where $w_{CR,j}^{k}$ represents the weight of criterion $CR_{j}$ satisfying that $w_{CR,j}^{k}\in \left[0,1\right]\left(k=1,2,...,p\right)$ and $\sum_{j=1}^{n}w_{CR,j}^{k}=1$. Let $O={\left(o_{1},o_{2},...,o_{p}\right)}^{T}$ be the weight vector of the Eps, where $o_{k}$ is the weight of Ep satisfying that $o_{k}\in \left[0,1\right]\left(k=1,2,...,p\right)$ and $\sum_{k=1}^{p}o_{k}=1$.

Both $W_{CR}^{k}={\left(w_{CR,1}^{k},w_{CR,2}^{k},...,w_{CR,n}^{k}\right)}^{T}$ and $O={\left(o_{1},o_{2},...,o_{p}\right)}^{T}$ are unknown, and need to be determined according to the decision information. The evaluation values of Eps on the program attributes are expressed by TIFNs. For example, the evaluation value of Ep $DM_{k}$ on attribute $CR_{j}$ of $B_{i}$ is a TIFN ${\tilde{a}}_{ij}^{k}=\left(\left({\underline{a}}_{ij}^{k},a_{ij}^{k},{\bar{a}}_{ij}^{k}\right);\omega_{ij}^{k},u_{ij}^{k}\right)$. Thus, the problems with TIFNs can be expressed as multiattribute decision matrix ${\tilde{N}}^{k}={\left({\tilde{n}}_{ij}^{k}\right)}_{m\times n}\left(k=1,2,...,p\right)$.

***Phase 1**. Determining the weight of the criteria (BWM-Entropy Weight Method).*

BWM was proposed by Rezaei in 2015 [7]. This method simplifies the comparison process by selecting the best and the worst criteria among many, thus reducing the risk of inconsistency and reaching the accuracy of the judgment results. This method cannot fully reflect the information in the real data; the entropy weight method as a method to calculate the objective weight makes up for this deficiency. So, the two methods in this paper are combined to form a new I-BWM. The specific steps are as follows:

Step 1. Determine the criteria system.

The system is the basis for evaluating alternatives; suppose there is n criteria $CR_{j}(j=1,2,...,n)$ and $CR_{n}$ represents the nth criterion.

Step 2. Determine the best and worst criteria.

In this step, the best and worst criteria are determined by the Eps, whereby $CR_{b}$ represents the best criteria and $CR_{w}$ represents the worst.

Step 3. Compare the best and worst criteria with other criteria.

This step is divided into two parts: one is that the language variables in Table 1 are used by Eps to determine the preference of the best criterion over all other criteria. The best-to-others vector would be $\chi_{b}=\left({\tilde{x}}_{b1},{\tilde{x}}_{b2},...,{\tilde{x}}_{bn}\right)$, and the other is to determine the preference of the worst criterion over all other criteria. Similarly, the best-to-others vector would be $\chi_{w}=\left({\tilde{x}}_{1w},{\tilde{x}}_{2w},...,{\tilde{x}}_{nw}\right)$.

Step 4. The mathematical model will be established to calculate the target weight.

The aim is to calculate the optimal weights of criteria in this step. For the mathematical programming models, the optimal absolute difference is expressed as |w˜CR,bw˜CR,j−x˜bj|. The worst absolute difference |w˜CR,jw˜CR,w−x˜jw|, ϕ represents a consistency ratio, and ${\tilde{w}}_{CR,b}$, ${\tilde{w}}_{CR,j}$, ${\tilde{w}}_{CR,w}$ represent the weight of the criteria $CR_{b}$, $CR_{j}$, $CR_{w}$ respectively. The target weight can be obtained by Equation (6).

(7) minϕ{|w˜CR,bw˜CR,j−x˜bj|≤ϕ for all j|w˜CR,jw˜CR,w−x˜jw|≤ϕ for all j∑k=1nwCR,j=1wCR,j≥0

In the traditional BWM method, 1–9 is usually used as the evaluation criteria to select the best and worst criteria according to the subjective attitude of experts, which is not science. Therefore, TIFNs is introduced to replace the 1–9 scale, which can more accurately express the attitude of Eps. In this way, the above Equation (6) evolves into Equation (7).

(8) $$\begin{array}{l} \min\phi^{*}\\ s.t.\{\begin{array}{c} \left|\frac{\left({\underline{a}}_{CR,b},a_{CR,b},{\bar{a}}_{CR,b};\omega_{CR,b},u_{CR,b}\right)}{\left({\underline{a}}_{CR,j},a_{CR,j},{\bar{a}}_{CR,j};\omega_{CR,j},u_{CR,j}\right)}-\left({\underline{x}}_{bj},x_{bj},{\bar{x}}_{bj},w_{bj},u_{bj}\right)\right|\leq \phi^{*}\\ \left|\frac{\left({\underline{a}}_{CR,j},a_{CR,j},{\bar{a}}_{CR,j};\omega_{CR,j},u_{CR,j}\right)}{\left({\underline{a}}_{CR,w},a_{CR,w},{\bar{a}}_{CR,w};\omega_{CR,w},u_{CR,w}\right)}-\left({\underline{x}}_{jw},x_{jw},{\bar{x}}_{jw},\omega_{jw},u_{jw}\right)\right|\leq \phi^{*}\\ \sum_{k=1}^{n}R\left({\tilde{w}}_{CR,j}\right)=1\\ \begin{array}{c} {\underline{a}}_{CR,j}\leq a_{CR,j}\leq {\bar{a}}_{CR,j}\\ \omega_{CR,j}\leq u_{CR,j}\\ {\tilde{w}}_{CR,j}\geq 0\\ j=1,2,...,n \end{array} \end{array} \end{array}$$

Next, supposed that:

(9) $$\begin{array}{l} \left|\frac{\left({\underline{a}}_{CR,b},a_{CR,b},{\bar{a}}_{CR,b};\omega_{CR,b},u_{CR,b}\right)}{\left({\underline{a}}_{CR,j},a_{CR,j},{\bar{a}}_{CR,j};\omega_{CR,j},u_{CR,j}\right)}-\left({\underline{x}}_{bj},x_{bj},{\bar{x}}_{bj},w_{bj},u_{bj}\right)\right|=\alpha ,\\ \left|\frac{\left({\underline{a}}_{CR,j},a_{CR,j},{\bar{a}}_{CR,j};\omega_{CR,j},u_{CR,j}\right)}{\left({\underline{a}}_{CR,w},a_{CR,w},{\bar{a}}_{CR,w};\omega_{CR,w},u_{CR,w}\right)}-\left({\underline{x}}_{jw},x_{jw},{\bar{x}}_{jw},\omega_{jw},u_{jw}\right)\right|=\beta \end{array}$$

(1) When α>0, β>0

(10) $$\begin{array}{l} \min\phi^{*}\\ s.t.\{\begin{array}{c} \frac{\left({\underline{a}}_{CR,b},a_{CR,b},{\bar{a}}_{CR,b};\omega_{CR,b},u_{CR,b}\right)}{\left({\underline{a}}_{CR,j},a_{CR,j},{\bar{a}}_{CR,j};\omega_{CR,j},u_{CR,j}\right)}-\left({\underline{x}}_{bj},x_{bj},{\bar{x}}_{bj},w_{bj},u_{bj}\right)\leq \phi^{*}\\ \frac{\left({\underline{a}}_{CR,j},a_{CR,j},{\bar{a}}_{CR,j};\omega_{CR,j},u_{CR,j}\right)}{\left({\underline{a}}_{CR,w},a_{CR,w},{\bar{a}}_{CR,w};\omega_{CR,w},u_{CR,w}\right)}-\left({\underline{x}}_{jw},x_{jw},{\bar{x}}_{jw},\omega_{jw},u_{jw}\right)\leq \phi^{*}\\ \sum_{k=1}^{n}R\left({\tilde{w}}_{CR,j}\right)=1\\ \begin{array}{c} {\underline{a}}_{CR,j}\leq a_{CR,j}\leq {\bar{a}}_{CR,j}\\ \omega_{CR,j}\leq u_{CR,j}\\ {\tilde{w}}_{CR,j}\geq 0\\ j=1,2,...,n \end{array} \end{array} \end{array}$$

(2) When α<0, β<0

(11) $$\begin{array}{l} \min\phi^{*}\\ s.t.\{\begin{array}{c} \left({\underline{x}}_{bj},x_{bj},{\bar{x}}_{bj},w_{bj},u_{bj}\right)-\frac{\left({\underline{a}}_{CR,b},a_{CR,b},{\bar{a}}_{CR,b};\omega_{CR,b},u_{CR,b}\right)}{\left({\underline{a}}_{CR,j},a_{CR,j},{\bar{a}}_{CR,j};\omega_{CR,j},u_{CR,j}\right)}\leq \phi^{*}\\ \left({\underline{x}}_{jw},x_{jw},{\bar{x}}_{jw},\omega_{jw},u_{jw}\right)-\frac{\left({\underline{a}}_{CR,j},a_{CR,j},{\bar{a}}_{CR,j};\omega_{CR,j},u_{CR,j}\right)}{\left({\underline{a}}_{CR,w},a_{CR,w},{\bar{a}}_{CR,w};\omega_{CR,w},u_{CR,w}\right)}\leq \phi^{*}\\ \sum_{k=1}^{n}R\left({\tilde{w}}_{CR,j}\right)=1\\ \begin{array}{c} {\underline{a}}_{CR,j}\leq a_{CR,j}\leq {\bar{a}}_{CR,j}\\ \omega_{CR,j}\leq u_{CR,j}\\ {\tilde{w}}_{CR,j}\geq 0\\ j=1,2,...,n \end{array} \end{array} \end{array}$$

(3) When α>0, β<0

(12) $$\begin{array}{l} \min\phi^{*}\\ s.t.\{\begin{array}{c} \frac{\left({\underline{a}}_{CR,b},a_{CR,b},{\bar{a}}_{CR,b};\omega_{CR,b},u_{CR,b}\right)}{\left({\underline{a}}_{CR,j},a_{CR,j},{\bar{a}}_{CR,j};\omega_{CR,j},u_{CR,j}\right)}-\left({\underline{x}}_{bj},x_{bj},{\bar{x}}_{bj},w_{bj},u_{bj}\right)\leq \phi^{*}\\ \left({\underline{x}}_{jw},x_{jw},{\bar{x}}_{jw},\omega_{jw},u_{jw}\right)-\frac{\left({\underline{a}}_{CR,j},a_{CR,j},{\bar{a}}_{CR,j};\omega_{CR,j},u_{CR,j}\right)}{\left({\underline{a}}_{CR,w},a_{CR,w},{\bar{a}}_{CR,w};\omega_{CR,w},u_{CR,w}\right)}\leq \phi^{*}\\ \sum_{k=1}^{n}R\left({\tilde{w}}_{CR,j}\right)=1\\ \begin{array}{c} {\underline{a}}_{CR,j}\leq a_{CR,j}\leq {\bar{a}}_{CR,j}\\ \omega_{CR,j}\leq u_{CR,j}\\ {\tilde{w}}_{CR,j}\geq 0\\ j=1,2,...,n \end{array} \end{array} \end{array}$$

(4) When α<0, β>0

(13) $$\begin{array}{l} \min\phi^{*}\\ s.t.\{\begin{array}{c} \left({\underline{x}}_{bj},x_{bj},{\bar{x}}_{bj},w_{bj},u_{bj}\right)-\frac{\left({\underline{a}}_{CR,b},a_{CR,b},{\bar{a}}_{CR,b};\omega_{CR,b},u_{CR,b}\right)}{\left({\underline{a}}_{CR,j},a_{CR,j},{\bar{a}}_{CR,j};\omega_{CR,j},u_{CR,j}\right)}\leq \phi^{*}\\ \frac{\left({\underline{a}}_{CR,j},a_{CR,j},{\bar{a}}_{CR,j};\omega_{CR,j},u_{CR,j}\right)}{\left({\underline{a}}_{CR,w},a_{CR,w},{\bar{a}}_{CR,w};\omega_{CR,w},u_{CR,w}\right)}-\left({\underline{x}}_{jw},x_{jw},{\bar{x}}_{jw},\omega_{jw},u_{jw}\right)\leq \phi^{*}\\ \sum_{k=1}^{n}R\left({\tilde{w}}_{CR,j}\right)=1\\ \begin{array}{c} {\underline{a}}_{CR,j}\leq a_{CR,j}\leq {\bar{a}}_{CR,j}\\ \omega_{CR,j}\leq u_{CR,j}\\ {\tilde{w}}_{CR,j}\geq 0\\ j=1,2,...,n \end{array} \end{array} \end{array}$$

The target weight value $\left(w_{CR,1}^{*},w_{CR,2}^{*},...,w_{CR,n}^{*}\right)$ can be obtained by solving Equation (7). The consistency index obtained must not exceed the maximum possible CI. The maximum possible CI for different linguistic variables of fuzzy TIFNs-BWM is listed in the Table 1.

As for the minimum consistency ${\tilde{x}}_{bn}={\tilde{x}}_{nw}={\tilde{x}}_{bw}$,

(14) $$\begin{array}{l} \left({\tilde{x}}_{bj}-\phi \right)\times \left({\tilde{x}}_{jw}-\phi \right)=\left({\tilde{x}}_{bw}+\phi \right)\\ \Rightarrow \left({\tilde{x}}_{bj}-\phi \right)\times \left({\tilde{x}}_{bj}-\phi \right)=\left({\tilde{x}}_{bk}+\phi \right)\\ \Rightarrow \phi^{2}-\left(1+2{\tilde{x}}_{bj}\right)\phi +\left({\tilde{x}}_{bj}{}^{2}-{\tilde{x}}_{bj}\right)=0 \end{array}$$

The consistency ratio can be calculated by Consistency Ratio=ϕ*CI. The closer to 0, the better the consistency. Complete consistency is achieved when CI is zero.

**Figure 5:**
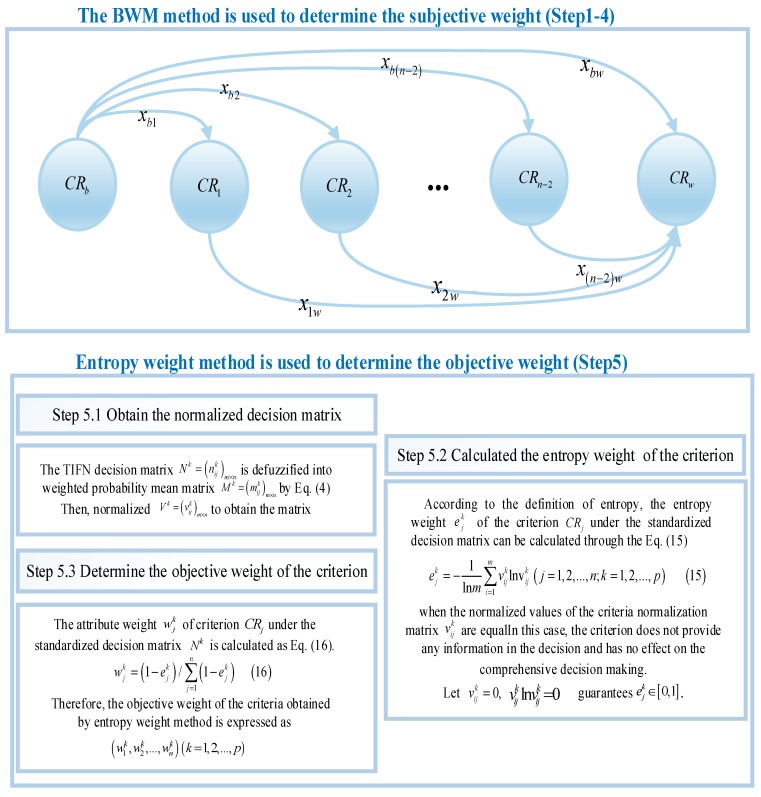
Objective weight calculation steps.

Step 5. The entropy weight method is used to determine the objective weight. The specific process and the releated Equations (15) and (16) are shown in Figure 5.

(15) $$e_{j}^{k}=-\frac{1}{\mathrm{lnm}}\sum_{i=1}^{m}v_{ij}^{k}\ln v_{ij}^{k}\left(j=1,2,\ldots ,n,k=1,2,\ldots ,p\right)$$

(16) $$w_{j}^{k}=\left(1-e_{j}^{k}\right)/\sum_{j=1}^{n}\left(1-e_{j}^{k}\right)$$

Step 6. The entropy weight method is used to modify BWM.

In order to combine the BWM and the entropy weight methods, the entropy value variable is introduced and the final weight value $w_{CR,j}^{*}$ is determined by Equation (17).

(17) $$w_{CR,j}^{k}=w_{CR,j}^{*}e_{j}^{k}+w_{j}^{k}\left(1-e_{j}^{k}\right),\left(j=1,2,...,n\right)$$

Where $w_{CR,j}^{*}$ is the weight value determined by BWM method and $w_{j}^{k}$ is the weight value determined by the entropy weight method. To ensure the final weight, the value must conform to the following two properties:

(1) The final determined weight should be between the weights determined by the two methods.

(2) The entropy value $e_{j}^{k}$ is relatively large: $\left(e_{j}^{k}>0.5\right)$, $w_{CR,n}^{k}$ is closer to $w_{CR,j}^{*}$ and $e_{j}^{k}$ is relatively small $\left(e_{j}^{k}<0.5\right)$, $w_{CR,n}^{k}$ is closer to $w_{j}^{k}$.

***Phase 2**. B-DST is used to determine the weights of Eps.*

B-DST is an extension of DST, which can be used to deal with the uncertainty existing in things. It decomposes the complex large evidence into simple, small pieces of evidence in a certain way. After the relevant processing of small evidence, it uses combination rules to synthesize the processing results, and finally, obtains the solution to the problem [48,49]. In this part, a Bayes approximation method based on Dempster’s rule of evidence synthesis is used as the basis to obtain the weights of Eps.

For the TIFN multiattribute group decision matrix ${\tilde{N}}^{k}={\left({\tilde{n}}_{ij}^{k}\right)}_{m\times n}$ and normalized weighted probability mean matrix $V^{k}={\left(v_{ij}^{k}\right)}_{m\times n}$, let $\psi =\left\{B_{1},B_{2},...,B_{m}\right\}$ be used as the identification framework. $B_{i}$ indicates the ith plan. All criteria values in $V^{k}$ are the evidence body of the criteria. In other words, for the evaluation value of $DM_{k}$ on criteria attribute $a_{i}$ of plan $B_{i}$, $v_{ij}^{k}$ is the evidence body $m_{j}^{k}\left(A_{i}\right)$, as shown in Equation (18).

(18) $$m_{j}^{k}\left(B_{i}\right){=v}_{ij}^{k}\;\left(i=1,2,...,m;\;j=1,2,...,n;\;k=1,2,...,p\right)$$

Step 1. Determine the attribute weighted evidence body.

In the previous phase, the final criteria attribute weight modified by the entropy weight method to BWM is $w_{CR,j}^{k}$, and the attribute weight evidence body ${m^{\prime }}_{j}^{k}\left(B_{i}\right)$ of scheme $B_{i}$ under $CR_{j}$ could be obtained by Equations (19) and (20), as shown in the matrix $C^{*}$.

(19) $${m^{\prime }}_{j}^{k}\left(B_{i}\right)=w_{CR,j}^{k}m_{j}^{k}\left(B_{i}\right)$$

(20) $${m^{\prime }}_{j}^{k}\left(\psi \right)=1-\sum_{i=1}^{m}{m^{\prime }}_{j}^{k}\left(B_{i}\right)=1-w_{CR,n}^{k}\sum_{i=1}^{m}m_{j}^{k}\left(B_{i}\right)$$

(21) C*=[DM1m′j1(Bi)CR1CR2…CRnB1wCR,11m11(B1)wCR,21m21(B1)…wCR,n1mn1(B1)B2wCR,11m11(B2)wCR,21m21(B2)…wCR,n1mn1(B2)⋮⋮⋮⋮⋮BmwCR,11m11(Bm)wCR,21m21(Bm)…wCR,n1mn1(B3)ψ1−wCR,11∑i=1mm11(Bi)1−wCR,21∑i=1mm21(Bi)…1−wCR,n1∑i=1mmn1(Bi)⋯DMnm′jn(Bi)CR1CR2…CRnB1wCR,1nm1n(B1)wCR,2nm2n(B1)…wCR,nnmnn(B1)B2wCR,1nm1n(B2)wCR,2nm2n(B2)…wCR,nnmnn(B2)⋮⋮⋮⋮⋮BmwCR,1nm1n(Bm)wCR,2nm2n(Bm)…wCR,nnmnn(B3)ψ1−wCR,1n∑i=1mm1n(Bi)1−wCR,2n∑i=1mm2n(Bi)…1−wCR,nn∑i=1mmnn(Bi)]

Step 2. Calculate the Bayes approximation function value of the attribute weighted evidence body.

By considering the credibility of the criteria in the weighted evidence body, the uncertainty information provided by the criterion evidence with a low level of credibility will be reduced, and the uncertainty information provided by the uncertain criterion element will be increased, thereby reducing the influence of the criterion evidence body with low levels of credibility on the whole decision result.

The Bayes approximation function value ${\underline{m^{\prime }}}_{j}^{k}\left(B_{i}\right)$ of attribution-weighted evidence body ${m^{\prime }}_{j}^{k}\left(B_{i}\right)$ is calculated by Equation (22).

(22) $${\underline{m^{\prime }}}_{j}^{k}\left(B_{i}\right)=\frac{w_{CR,1}^{k}m_{1}^{k}\left(B_{1}\right)+\left(1-w_{CR,1}^{k}\sum_{i=1}^{m}m_{1}^{k}\left(B_{i}\right)\right)}{w_{CR,1}^{k}m_{1}^{k}\left(B_{1}\right)\times 1+w_{CR,1}^{k}m_{1}^{k}\left(B_{2}\right)\times 1+\cdots +w_{CR,1}^{k}m_{1}^{k}\left(B_{m}\right)\times 1+\left(1-w_{CR,1}^{k}\sum_{i=1}^{m}m_{1}^{k}\left(B_{i}\right)\right)\times m}$$

Step 3. Determine the weight of comprehensive evidence.

According to the Bayes approximate formula of evidence theory, the attribution weighted evidence body ${m^{\prime }}_{j}^{k}\left(B_{i}\right)$ can be fused into comprehensive evidence weight body ${m^{\prime }}_{j}^{k}\left(B_{i}\right)$, which can be calculated by Equation (23).

(23) $${\underline{m}}^{k}\left(B_{i}\right)=\frac{\left[{\underline{m}}_{1}^{1}(B_{1})\times {\underline{m}}_{2}^{1}(B_{1})\times \cdots \times {\underline{m}}_{n}^{1}(B_{1})\right]}{\left[{\underline{m}}_{1}^{k}(B_{1})\times \cdots \times {\underline{m}}_{n}^{k}(B_{1})\right]+\left[{\underline{m}}_{1}^{k}(B_{2})\times \cdots \times {\underline{m}}_{n}^{k}(B_{2})\right]+\cdots +\left[{\underline{m}}_{1}^{k}(B_{m})\times \cdots \times {\underline{m}}_{n}^{k}(B_{m})\right]}$$

where comprehensive weighted evidence body ${\underline{m}}_{}^{k}\left(B_{i}\right)$ represents $DM_{k}$ individual evaluation evidence of scheme $B_{i}$.

Step 4. Calculate the distance and similarity between the sets of evidence.

Let evidence set ${\underline{m}}_{}^{k}$ be a set of comprehensive weighted evidence ${\underline{m}}_{}^{k}\left(B_{i}\right)$ (i=1,2,...,m) for any set of evidence ${\underline{m}}_{}^{q}$ and ${\underline{m}}_{}^{k}$; the distance between them is calculated as Equation (24)

(24) $$d\left({\underline{m}}_{}^{q},{\underline{m}}_{}^{t}\right)=\sqrt{\frac{\left[M_{}^{q},M^{q}\right]+\left[M_{}^{t},M^{t}\right]-2\left[M_{}^{t},M_{}^{q}\right]}{2}}\left(q,t\in k\right)$$

where $M_{}^{k}=\left({\underline{m}}^{k}\left(B_{1}\right),\;{\underline{m}}^{k}\left(B_{2}\right),...,{\underline{m}}^{k}\left(B_{m}\right)\right)\;\left(k=q,t\right)$, $\left[M_{}^{q},M_{}^{t}\right]$ is defined as $\left[M_{}^{q},M_{}^{t}\right]=\sum_{i=1}^{z}\sum_{j=1}^{z}\left[{\underline{m}}^{q}\left(B_{i}\right){\underline{m}}^{t}\left(B_{j}\right)d_{ij}\right]$, and $d_{ij}=\frac{\left|B_{i}\cap B_{j}\right|}{\left|B_{i}\cup B_{j}\right|}\left(i,j=1,...,2^{z}\right)$.

Similarity $d\left({\underline{m}}_{}^{q},{\underline{m}}_{}^{k}\right)$ and distance $s\left({\underline{m}}_{}^{q},{\underline{m}}_{}^{k}\right)$ are a pair of opposite concepts. The smaller the distance of the evidence body, the greater their similarity. The similarity between ${\underline{m}}_{}^{q}$ and ${\underline{m}}_{}^{k}$ can be calculated by Equations (25)–(27).

(25) $$s\left({\underline{m}}_{}^{q},{\underline{m}}_{}^{k}\right)=1-d\left({\underline{m}}_{}^{q},{\underline{m}}_{}^{k}\right)\;\left(q,t=1,2,...,p\right)$$

(26) $$D_{M}={\left(d\left({\underline{m}}_{}^{q},{\underline{m}}_{}^{k}\right)\right)}_{m\times m}=\begin{array}{cc} & \begin{array}{ccccc} {\underline{m}}^{1} & {\underline{m}}^{2} & {\underline{m}}^{3} & \cdots & {\underline{m}}^{m} \end{array}\\ \begin{array}{c} {\underline{m}}^{1}\\ {\underline{m}}^{2}\\ \begin{array}{c} {\underline{m}}^{3}\\ \vdots \end{array}\\ {\underline{m}}^{m} \end{array} & {\left[\begin{array}{ccccc} 0 & d\left({\underline{m}}_{}^{1},{\underline{m}}_{}^{2}\right) & d\left({\underline{m}}_{}^{1},{\underline{m}}_{}^{3}\right) & \cdots & d\left({\underline{m}}_{}^{1},{\underline{m}}_{}^{m}\right)\\ d\left({\underline{m}}_{}^{1},{\underline{m}}_{}^{2}\right) & 0 & d\left({\underline{m}}_{}^{2},{\underline{m}}_{}^{3}\right) & \cdots & d\left({\underline{m}}_{}^{2},{\underline{m}}_{}^{m}\right)\\ d\left({\underline{m}}_{}^{1},{\underline{m}}_{}^{3}\right) & d\left({\underline{m}}_{}^{2},{\underline{m}}_{}^{3}\right) & 0 & \cdots & d\left({\underline{m}}_{}^{3},{\underline{m}}_{}^{m}\right)\\ \vdots & \vdots & \vdots & \vdots & \vdots \\ d\left({\underline{m}}_{}^{1},{\underline{m}}_{}^{m}\right) & d\left({\underline{m}}_{}^{2},{\underline{m}}_{}^{m}\right) & d\left({\underline{m}}_{}^{3},{\underline{m}}_{}^{m}\right) & \cdots & 0 \end{array}\right]}_{m\times m} \end{array}$$

(27) $$S_{M}={\left(s\left({\underline{m}}_{}^{q},{\underline{m}}_{}^{k}\right)\right)}_{m\times m}=\begin{array}{cc} & \begin{array}{cccc} {\underline{m}}^{1} & {\underline{m}}^{2} & \cdots & {\underline{m}}^{m} \end{array}\\ \begin{array}{c} {\underline{m}}^{1}\\ {\underline{m}}^{2}\\ \begin{array}{c} {\underline{m}}^{3}\\ \vdots \end{array}\\ {\underline{m}}^{m} \end{array} & {\left[\begin{array}{cccc} 1 & 1-d\left({\underline{m}}_{}^{1},{\underline{m}}_{}^{2}\right) & \cdots & 1-d\left({\underline{m}}_{}^{1},{\underline{m}}_{}^{m}\right)\\ 1-d\left({\underline{m}}_{}^{1},{\underline{m}}_{}^{2}\right) & 1 & \cdots & 1-d\left({\underline{m}}_{}^{2},{\underline{m}}_{}^{m}\right)\\ 1-d\left({\underline{m}}_{}^{1},{\underline{m}}_{}^{3}\right) & 1-d\left({\underline{m}}_{}^{2},{\underline{m}}_{}^{3}\right) & \cdots & 1-d\left({\underline{m}}_{}^{3},{\underline{m}}_{}^{m}\right)\\ \vdots & \vdots & \vdots & \vdots \\ 1-d\left({\underline{m}}_{}^{1},{\underline{m}}_{}^{m}\right) & 1-d\left({\underline{m}}_{}^{2},{\underline{m}}_{}^{m}\right) & \cdots & 1 \end{array}\right]}_{m\times m} \end{array}$$

Step 5. Calculate the weight of Eps.

If the similarity between the evidence is relatively large, it can be considered that the degree of mutual support between the evidence is relatively high, that is, the evidences are mutually supportive. In general, the higher the degree to which an evidence is supported by other evidence, the more credible that evidence. Let $Sup\left({\underline{m}}_{}^{k}\right)$ represents the support degree of other pieces of evidence to evidence ${\underline{m}}_{}^{k}$. The support degree function is calculated using Equation (28).

Let the relative confidence $crd\left({\underline{m}}_{}^{k}\right)$ of ${\underline{m}}_{}^{k}$ be treated as the weight $v_{k}$ of ${\underline{m}}_{}^{k}$.The weight vector of the evidence is expressed as $O={\left(o_{1},o_{2},...,o_{p}\right)}^{T}$, which is obtained using Equation (29).

(28) $$\mathrm{Sup}\left({\underline{m}}_{}^{k}\right)=\sum_{t=1,t\neq k}^{p}s\left({\underline{m}}_{}^{q},{\underline{m}}_{}^{k}\right)\;\left(k=1,2,...,p\right)$$

(29) $$o_{k}=crd\left({\underline{m}}_{}^{k}\right)/\sum_{t=1}^{p}crd\left({\underline{m}}_{}^{k}\right)$$

***Phase 3** Determine the ranking of suppliers and select the best LSSs.*

VIKOR is an optimal compromise solution sequencing method based on ideal solutions which determines the positive ideal solution and negative ideal solution of the decision matrix, and then sorts the solution according to the proximity between the attribute evaluation value of each alternative and the ideal solution. It can consider both group utility maximization and individual regret minimization, and fully reflect the subjective preference of decision makers. On the basis of the above studies and the classical VIKOR, this paper proposes an extension method of TIFNs multiattribute group decision making to select the optimal LSS. The main steps are as follows:

Step 1. Calculate the attribute weight vector of comprehensive criteria.

According to the research on criteria attribute weights $W_{CR}^{k}={\left(w_{CR,1}^{k},w_{CR,2}^{k},...,w_{CR,n}^{k}\right)}^{T},\;\left(k=1,2,...p\right)$ in phase 1 and Eps weight $O={\left(o_{1},o_{2},...,o_{p}\right)}^{T}$ in phase 2, Equation (30) can be used to calculate the comprehensive criteria attribute weight.

(30) $$w_{j}=\sum_{k=1}^{p}o_{k}w_{CR,j}^{k}\;(j=1,2,...,n)$$

Step 2. Construct the comprehensive decision matrix.

According to the TIF-WAA operator in Definition 3, the following Equations (31) and (32) are used to integrate a single decision matrix ${\tilde{N}}^{k}={\left({\tilde{n}}_{ij}^{k}\right)}_{m\times n}\left(k=1,2,...,p\right)$ into a comprehensive weighted matrix $\tilde{Z}={\left({\tilde{z}}_{ij}^{}\right)}_{m\times n}$.

(31) $$\tilde{Z}={\left({\tilde{z}}_{ij}^{}\right)}_{m\times n}=\begin{array}{cc} & \begin{array}{ccccc} CR_{1} & CR_{2} & CR_{3} & \cdots & CR_{n} \end{array}\\ \begin{array}{c} B_{1}\\ B_{2}\\ B_{3}\\ \vdots \\ B_{m} \end{array} & \left[\begin{array}{ccccc} {\tilde{z}}_{11} & {\tilde{z}}_{12} & {\tilde{z}}_{13} & \ldots & {\tilde{z}}_{1n}\\ {\tilde{z}}_{21} & {\tilde{z}}_{22} & {\tilde{z}}_{23} & \ldots & {\tilde{z}}_{2n}\\ {\tilde{z}}_{31} & {\tilde{z}}_{32} & {\tilde{z}}_{33} & \ldots & {\tilde{z}}_{3n}\\ \vdots & \vdots & \vdots & \vdots & \vdots \\ {\tilde{z}}_{m1} & {\tilde{z}}_{m2} & {\tilde{z}}_{m3} & \ldots & {\tilde{z}}_{mn} \end{array}\right] \end{array}$$

(32) $$\begin{array}{l} {\tilde{z}}_{ij}^{}=\left(\left(z_{1i}\left(n_{j}\right),z_{2i}\left(n_{j}\right),z_{3i}\left(n_{j}\right)\right);\omega_{z_{ij}},u_{z_{ij}}\right)=\mathrm{TIF}-\mathrm{WAA}\left({\tilde{n}}_{ij}^{1},{\tilde{n}}_{ij}^{2},...,{\tilde{n}}_{ij}^{p}\right)\\ =\sum_{k=1}^{p}o_{k}{\tilde{n}}_{ij}^{k}=\left(\left(\sum_{k=1}^{p}o_{k}{\underline{n}}_{ij}^{k},\sum_{k=1}^{p}o_{k}n_{ij}^{k},\sum_{k=1}^{p}o_{k}{\bar{n}}_{ij}^{k}\right);\underset{1\leq k\leq p}{\min}\left\{\omega_{n_{ij}}^{k}\right\},\max\left\{u_{n_{ij}}^{k}\right\}\right) \end{array}$$

Step3. Determine the positive and negative ideal solutions, where the positive ideal solution is $B^{+}=\left\{{\tilde{b}}_{1}^{+},{\tilde{b}}_{2}^{+},...,{\tilde{b}}_{n}^{+}\right\}$ and the negative ideal solution is $B^{-}=\left\{{\tilde{b}}_{1}^{-},{\tilde{b}}_{2}^{-},...,{\tilde{b}}_{n}^{-}\right\}$.

(33) $${\tilde{b}}^{+}=\left(\left(\underset{1\leq i\leq m}{\max}\left\{z_{1i}\left(n_{j}\right)\right\}\right),\left(\underset{1\leq i\leq m}{\max}\left\{z_{2i}\left(n_{j}\right)\right\}\right),\left(\underset{1\leq i\leq m}{\max}\left\{z_{3i}\left(n_{j}\right)\right\}\right);1,0\right)$$

(34) $${\tilde{b}}^{-}=\left(\left(\underset{1\leq i\leq m}{\min}\left\{z_{1i}\left(n_{j}\right)\right\}\right),\left(\underset{1\leq i\leq m}{\min}\left\{z_{2i}\left(n_{j}\right)\right\}\right),\left(\underset{1\leq i\leq m}{\min}\left\{z_{3i}\left(n_{j}\right)\right\}\right);0,1\right)$$

Step 4. Calculate the group utility values, the individual regret values for each alternative, and the approximation between the alternative solution and the ideal solution.

The Hamming distance of TIFN in Definition 4 is used in Equations (35) and (36) to calculate the above group utility value $S\left(B_{i}\right)$ and individual regret value $R\left(B_{i}\right)$.

(35) $$\begin{array}{l} S\left(B_{i}\right)=\sum_{j=1}^{n}w_{j}\\ \left\{\frac{\left(\left|\left(\underset{1\leq i\leq m}{\max}\left\{z_{1i}\left(n_{j}\right)\right\}\right)-\underline{z}\right|+\left|\left(\underset{1\leq i\leq m}{\max}\left\{z_{2i}\left(n_{j}\right)\right\}\right)-z\right|+\left|\left(\underset{1\leq i\leq m}{\max}\left\{z_{3i}\left(n_{j}\right)\right\}\right)-\bar{z}\right|+\max\left(\left|1-\omega_{\tilde{z}}\right|,\left|0-u_{\tilde{z}}\right|\right)\right)}{3\left(\left|\left(\underset{1\leq i\leq m}{\max}\left\{z_{1i}\left(n_{j}\right)\right\}\right)-\left(\underset{1\leq i\leq m}{\min}\left\{z_{1i}\left(n_{j}\right)\right\}\right)\right|+\left|\left(\underset{1\leq i\leq m}{\max}\left\{z_{2i}\left(n_{j}\right)\right\}\right)-\left(\underset{1\leq i\leq m}{\min}\left\{z_{2i}\left(n_{j}\right)\right\}\right)\right|+\left|\left(\underset{1\leq i\leq m}{\max}\left\{z_{3i}\left(n_{j}\right)\right\}\right)-\left(\underset{1\leq i\leq m}{\min}\left\{z_{3i}\left(n_{j}\right)\right\}\right)\right|+1\right)}\right\} \end{array}$$

(36) $$\begin{array}{l} R\left(B_{i}\right)=\underset{1\leq j\leq n}{\max}\\ \left\{w_{j}\frac{\left(\left|\left(\underset{1\leq i\leq m}{\max}\left\{z_{1i}\left(n_{j}\right)\right\}\right)-\bar{z}\right|+\left|\left(\underset{1\leq i\leq m}{\max}\left\{z_{2i}\left(n_{j}\right)\right\}\right)-z\right|+\left|\left(\underset{1\leq i\leq m}{\max}\left\{z_{3i}\left(n_{j}\right)\right\}\right)-\underline{z}\right|+\max\left(\left|1-\omega_{\tilde{c}}\right|,\left|0-u_{\tilde{c}}\right|\right)\right)}{3\left(\left|\left(\underset{1\leq i\leq m}{\max}\left\{z_{1i}\left(n_{j}\right)\right\}\right)-\left(\underset{1\leq i\leq m}{\min}\left\{z_{1i}\left(n_{j}\right)\right\}\right)\right|+\left|\left(\underset{1\leq i\leq m}{\max}\left\{z_{2i}\left(n_{j}\right)\right\}\right)-\left(\underset{1\leq i\leq m}{\min}\left\{z_{2i}\left(n_{j}\right)\right\}\right)\right|+\left|\left(\underset{1\leq i\leq m}{\max}\left\{z_{3i}\left(n_{j}\right)\right\}\right)-\left(\underset{1\leq i\leq m}{\min}\left\{z_{3i}\left(n_{j}\right)\right\}\right)\right|+1\right)}\right\} \end{array}$$

Calculate the approximation between the alternative solution and the ideal solution $Q\left(B_{i}\right)$ using Equation (37)

(37) $$Q\left(B_{i}\right)=\theta \frac{S\left(B_{i}\right)-S^{\max}}{S^{\min}-S^{\max}}+\left(1-\theta \right)\frac{R\left(B_{i}\right)-R^{\max}}{R^{\min}-R^{\max}}$$

where $S^{\max}=\underset{i}{\max}\left(S_{i}\right)$, $S^{\min}=\underset{i}{\min}\left(S_{i}\right)$, $R^{\max}=\underset{i}{\max}\left(R_{i}\right)$, and $R^{\min}=\underset{i}{\min}\left(R_{i}\right)$, θ represents the group utility maximization coefficient. This paper takes θ=0.5, which indicates that the decision result is determined by the majority for good evaluations and the minority for poor evaluations.

Step 5. The evaluation results are obtained by sorting the alternatives.

According to $Q\left(B_{i}\right)\left(i=1,2,...,m\right)$, the alternatives are ranked from small to large, so as to obtain the optimal one.

## 5. Case Analysis

In this section, five typical LSSs in area J are evaluated. Firstly, the background information of the five alternatives is given. Secondly, the proposed method was used to evaluate the five LSSs and determine the sorting order. Finally, the influence of different situations on the evaluation results was obtained through sensitivity and comparative analyses.

### 5.1. Background Information

In the context of global climate change, it is the common aspiration of mankind to reduce greenhouse gas emissions and achieve sustainable economic development. Low-carbon economy has become a trend of sustainable development for the global economy. As an important part of the global economy, tourism has an unshirkable responsibility to respond to the call for low-carbon development. As the carrier of tourism, the construction for LSSs is of incomparable significance. In recent years, various types of LSSs have been springing up, but a series of problems such as ununified selection subjects and incomplete construction methods have become increasingly prominent. Therefore, systematically evaluating LSSs is a problem that needs to be solved by academia. Based on the objective reality, and taking LSSs as the research object, this paper proposes a new MCDM to effectively solve the problem of LSS evaluation. 

### 5.2. Evaluate process and Results

There were three Eps $\left\{DM_{1},DM_{2},DM_{3}\right\}$ to evaluate five LSSs $\left\{B_{1},B_{2},B_{3},B_{4},B_{5}\right\}$. Each LSS is composed of twenty-two criteria $CR_{j}(j=1,2,...,22)$. Recommended by the competent department of tourist attractions and evaluated by experts on the spot in area J, the evaluator consisted of three panels, including tourism professors, scenic spot managers, and tourism-related government workers. According to Definition 6, the expert selected appropriate language variables to evaluate the five alternatives.

***Phase 1.** Determine the weight of the criteria.*

After consultation, the Eps determined the optimal criterion $CR_{1}$ and the worst criterion $CR_{11}$, and obtained the optimal comparison vector $\chi_{1}=\left(1,{\tilde{x}}_{1,2},...,{\tilde{x}}_{1,22}\right)$ and the worst comparison vector $\chi_{11}=\left({\tilde{x}}_{1,11},{\tilde{x}}_{2,11},...,{\tilde{x}}_{22,11}\right)$ by referring to the language variables in Table 1. According to Equations (7)–(13), the subjective weights of the 22 criteria can be obtained as follows:

$$\left(w_{CR,1}^{*},w_{CR,2}^{*},w_{CR,3}^{*},...,w_{CR,22}^{*}\right)={\left(\begin{array}{l} 0.0842,0.0385,0.0842,0.0436,0.0842,0.0385,0.0516,0.0430,0.0375,0.0334,0.0334,\\ 0.0400,0.0467,0.0430,0.0430,0.0334,0.0375,0.0334,0.0334,0.0400,0.0400,0.0375 \end{array}\right)}^{T}$$

The consistency index $\phi^{*}=\left(2.395,2.427,3.120,4.487,5.435,6.348\right)$ can be obtained by Equation (14). According to Equation (4), the initial value of five alternatives LSSs was defuzzied and normalized to obtain three sets of evaluation matrices, as shown in Table 3, Table 4 and Table 5. The subjective weight obtained by Equations (15)–(17) was modified by the objective weight obtained by the entropy weight method; the results were shown in Table 6.

**Phase 2.** Calculate the weights of Eps.

According to Equations (19)–(22), the Eps comprehensive weighted body ${\underline{m}}_{j}^{1}\left(B_{i}\right),{\underline{m}}_{j}^{2}\left(B_{i}\right),{\underline{m}}_{j}^{2}\left(B_{i}\right)$ of evidence for alternatives $B_{1},B_{2},B_{3},B_{4},B_{5}$ on criteria attributes are $CR_{1},CR_{2},...,CR_{22}$, as is shown in Table 7, Table 8 and Table 9. According to Equation (23), ${\underline{m}}_{j}^{1}\left(B_{i}\right),{\underline{m}}_{j}^{2}\left(B_{i}\right),{\underline{m}}_{j}^{2}\left(B_{i}\right)$ can be integrated into the individual comprehensive evidence body of the Eps, as shown in Table 10. The evidence body similarity matrix obtained from Equations (24)–(27), as is shown in Equation (38).

(38) $$S_{M}=\left(\begin{array}{rrr} 1.00000 & 0.36734 & 0.36737\\ 0.36734 & 1.00000 & 0.36732\\ 0.36737 & 0.36732 & 1.00000 \end{array}\right)$$

The weight $O={\left(0.333343,0.333321,0.333336\right)}^{T}$ for $DM_{1},DM_{2},DM_{3}$ is obtained by Equations (28)–(29).

***Phase 3.** Determine the ranking and select the best LSS.*

By combining the weights obtained by phase1 and phase2 through Equation (30), the final weights for the 22 criteria can be obtained as shown in Table 11. Through Equations (31) and (32), the evaluation information of three Eps can be integrated into a comprehensive decision matrix, as shown in Table 12. Using Equations (33)–(37), the group utility value $S\left(B_{i}\right)$ and individual regret value $R\left(B_{i}\right)$ of each scheme, as well as their proximity to the ideal solution $Q\left(B_{i}\right)$, are shown in Table 13, and the ranking results of the five alternative scenic spots can be obtained as follows: $B_{1}>B_{3}>B_{4}>B_{2}>B_{5}$. $B_{1}$ is the best alternative scenic area. Refer to Xu (2017) for θ=0.5; the transformation of the result corresponding to θ transformation will be discussed in the following section.

The method proposed in this paper can consider the maximum value of group utility, the minimum value of majority regret, and the minimum value of individual regret. In addition, the weights proportion of scoring Eps are taken into account effectively through Bayesian distribution. The decision system chooses the decision mechanism θ=0.5, meaning that the decision makers make different decisions according to the consensus reached through negotiation. Therefore, the method proposed in this paper is effective and flexible.

### 5.3. Discussion

Due to the different selection of θ, the results are different. In this case, a sensitivity analysis was conducted from the perspective of θ selection to explore the influence on the evaluation results of alternatives $B_{1},B_{2},B_{3},B_{4},B_{5}$, thus verifying the robustness of the evaluation results. On this basis, other comparable MCDM methods, including the single entropy weight method and BWM, are used to determine the weight of the first stage, so as to evaluate the LSSs. The results of different methods will illustrate the feasibility and rationality of the proposed method.

#### 5.3.1. Sensitive Analyses

**Figure 6:**
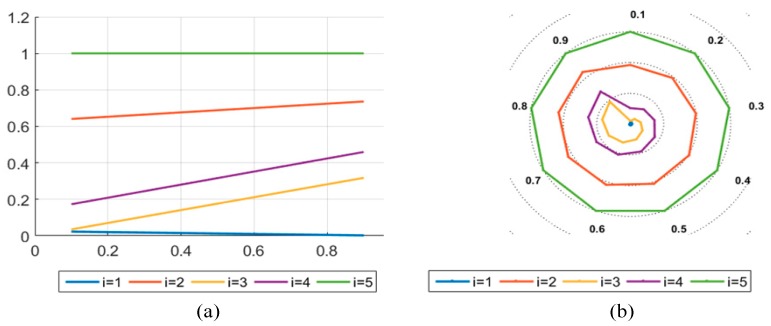
Sensitivity analysis results. (**a**) Description of what is the trend of the alternatives on the axis; (**b**) Description of what is the trend of the alternatives on the ring chart.

In this section, the impact of the group utility maximization coefficient θ on the results is discussed. θ<0.5 indicates that decision makers make decisions according to the decision mechanism that maximizes group utility; θ>0.5 indicates that decision makers make decisions according to the decision mechanism that minimizes individual regret. This paper assumes that θ=0.5, which indicates that decision makers make decisions according to the decision mechanism that reaches consensus through consultation. The value of θ is set to 0.1, 0.2, 0.3, 0.4, 0.5, 0.6, 0.7, 0.8, and 0.9 respectively. The results of the ideal solution approximation $Q\left(B_{i}\right)$ for the five alternatives and their rankings are shown in Table 14. In addition, in order to make the results more intuitive, two image representations were selected to express the final rankings. In Figure 6a, the abscissa represents the value of the θ, and the ordinate represents $Q\left(B_{i}\right)$ under different θ. In Figure 6b, the outermost circle represents θ and the lines of different species colors represent five LSSs; the left and right lines correspond in color. In the figures below, i = 1, 2, 3, 4, and 5 represent alternatives 1, 2, 3, 4, and 5. In addition, the left and right lines correspond to each other. As shown in Figure 6, when θ=0.1, the values of alternative scenic spot $B_{1}$ and $B_{3}$ are the closest, the gap between them becomes larger as θ increases, and the advantage of option $B_{1}$ becomes more obvious. $B_{5}$ is the most stable among all alternatives, and the change trends of option $B_{3}$ and $B_{4}$ are similar. The ranking is $B_{1}>B_{3}>B_{4}>B_{2}>B_{5}$. In summary, scenic spot $B_{1}$ is the optimal alternative.

#### 5.3.2. Comparative Analysis.

**Figure 7:**
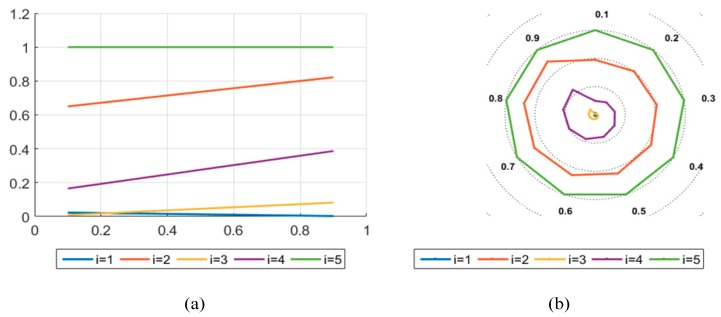
Result calculated by the entropy weight method. (**a**) Description of what is the trend of the alternatives on the axis; (**b**) Description of what is the trend of the alternatives on the ring chart.

**Figure 8:**
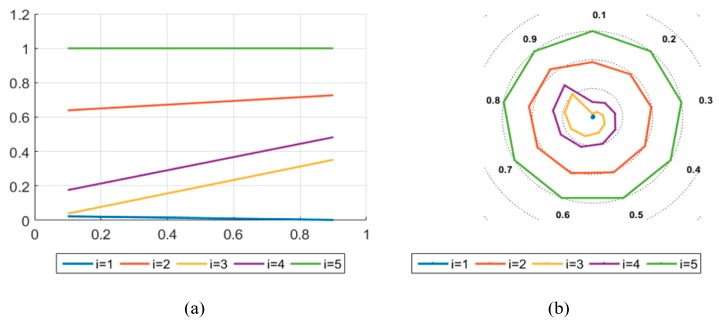
Result calculated by BWM. (**a**) Description of what is the trend of the alternatives on the axis; (**b**) Description of what is the trend of the alternatives on the ring chart.

In this paper, BWM is combined with the entropy weight method to obtain criteria weights. In order to demonstrate the scientific nature of the research results, this paper considers the comparison between the single BWM method and the single entropy weight method to calculate the weights. BWM is a very effective MCDM for determining criteria weights; it simplifies the operation process effectively by pairwise comparisons to calculate the required results. Entropy, as a measure of information uncertainty, can be used to determine the entropy weight of the criteria. The criteria weights are mainly determined according to the information contained in the evaluation value of the criteria, so as to avoid the influence of subjective factors. By comparison, the results obtained by the two methods are less different from those obtained by the research method in this paper. Therefore, the optimal multicriteria decision method of TIFNs proposed in this paper can effectively solve the MCDM problem. In comparison, the method in this paper considers both objective weight and subjective weight, and combines them effectively to make the evaluation results more universal. The evaluation results of the two methods are shown in Figure 7 and Figure 8. The results show that θ≠0.1,0.2, i.e., the result calculated by entropy weight method deviates slightly from other methods; for the alternative scenic spot, $B_{1}$ is the optimal LSS.

## 6. Conclusions

This paper proposes a new MCGDM method by which to study evaluation methods for LSSs. IBWM and B-DST are used to improve the traditional VIKOR method. Considering the uncertainty of the decision-making environment, TIFN was chosen as the evaluation language. Finally, the following conclusions are obtained.

(1) According to the comprehensive weight in Table 11, environmental factors and tourist facilities account for the largest proportion in the evaluation of LSSs. Therefore, build LSSs must carry out scientific planning. First of all, the construction of tourist attractions should use new technologies, new materials, and other energy-saving technologies to make the energy consumption levels of the scenic areas as low as possible; furthermore, it should prioritize integration and coordination with the surrounding ecological environment. Secondly, the number of tourists should be controlled in order to ensure low carbon emissions in the scenic area, according to reasonable limits. Third, the planning of scenic areas should attempt to increase green areas. Finally, the treatment of waste in the scenic area should be scientifically planned, e.g., by the separation and recovery of solid waste, wastewater and sewage treatment to be discharged into rivers, and so on.

(2) The operation of the scenic area is guided by low-carbon ideas. Scientific planning for tourist attractions is an important aspect of construction. The low-carbon concept should be put into every aspect of the operation of scenic spots, including strengthening the education of scenic area management personnel and service personnel. At the same time, scenic area managers should actively exchange and cooperate with foreign scenic spots to learn from their experience.

(3) LSSs should actively strengthen environmental protection education for tourists. Tourists are the main body of tourism activities, and their behavior has a direct impact on the carbon emissions of scenic spots. Therefore, it is necessary to actively strengthen environmental protection education for tourists.

The development of sustainable tourism and eco-tourism is a hot topic for experts and scholars at home and abroad. However, there are few research papers on low-carbon tourism. The method proposed in this paper can help select the best LSS and fill the academic gap in the LSS evaluation field. Then, in the context of global warming, this paper studies low-carbon evaluation criteria systems for tourist attractions. It is not uncommon for evaluation criteria systems to be applied in tourism, but this paper innovatively applies an evaluation system to the low-carbon evaluation of scenic spots. However, this research has some shortcomings. First, in the research process, the selection of the expert group members was relatively limited, and their views were relatively concentrated, which may have led to insufficient broadness of thought, i.e., making it too simple to reach consensus on issues. Second, the evaluation of alternative LSSs in this paper is expressed by TIFNs, and the evaluation value may be real numbers or other forms in practical application. Therefore, the MCGDM problem could be better studied when the decision information is of mixed language type.

## Figures and Tables

**Table 1:** Linguistic terms for fuzzy BWM.

Linguistic Term	TIFNs	Consistency Indices (CIs)
Equally Important(EI)	[(1,1,1;0.6), (1,1,1;0.3)]	2.395
Weakly Important(WI)	[(2/3,1,3/2;0.6), (2/3,2,3/2;0.3)]	2.427
Fairly Important(FI)	[(3/2,2,2/5;0.6), (3/2,2,2/5;0.3)]	3.120
Important(I)	[(5/2,3,7/2;0.6), (5/2,3,7/2;0.3)]	4.487
Very Important(VI)	[(7/2,4,9/2;0.6), [(7/2,4,9/2;0.3)]	5.435
Absolutely Important(AI)	[(9/2,5,11/2;0.6), (9/2,5,11/2;0.3)]	6.348

**Table 2:** Linguistic variables for experts, rating the relationship between alternatives and criteria.

Linguistic Term	TIFNs
Absolutely Low (AL)	[(0,0,1;0.6), (0,0,1;0.3)]
Low (L)	[(0,1,3;0.6), (0,1,3;0.3)]
Fairly Low (FL)	[(1,3,5;0.6), (1,3,5;0.3)]
Medium (M)	[(3,5,7;0.6), (3,5,7;0.3)]
Fairly High (FH)	[(5,7,9;0.6), [(5,7,9;0.3)]
High (H)	[(7,9,10;0.6), (7,9,10;0.3)]
Absolutely High (AH)	[(9,10,10;0.6), (9,10,10;0.3)]

**Table 3:** Evaluation matrix by $DM_{1}$.

	$B_{1}$	$B_{2}$	$B_{3}$	$B_{4}$	$B_{5}$
$CR_{1}$	0.1180	0.2449	0.2085	0.1966	0.2321
$CR_{2}$	0.2345	0.1427	0.2377	0.1998	0.1852
$CR_{3}$	0.2199	0.3089	0.1571	0.0366	0.2775
$CR_{4}$	0.2817	0.2988	0.0395	0.0395	0.3406
$CR_{5}$	0.0379	0.3781	0.2108	0.2108	0.1624
$CR_{6}$	0.2055	0.1468	0.2055	0.1468	0.2956
$CR_{7}$	0.1430	0.2287	0.2168	0.2168	0.1947
$CR_{8}$	0.1956	0.2177	0.1956	0.1956	0.1956
$CR_{9}$	0.1843	0.1981	0.1868	0.2327	0.1981
$CR_{10}$	0.2320	0.2576	0.0306	0.3057	0.1741
$CR_{11}$	0.2961	0.1676	0.0391	0.1676	0.3296
$CR_{12}$	0.2595	0.1749	0.1319	0.1749	0.2588
$CR_{13}$	0.2041	0.1832	0.2187	0.1970	0.1970
$CR_{14}$	0.0433	0.3397	0.0433	0.2460	0.3278
$CR_{15}$	0.1508	0.2111	0.2761	0.2111	0.1508
$CR_{16}$	0.0959	0.0959	0.2825	0.2120	0.3137
$CR_{17}$	0.2056	0.2056	0.1543	0.2289	0.2056
$CR_{18}$	0.2632	0.1778	0.1342	0.2370	0.1878
$CR_{19}$	0.1270	0.2244	0.2558	0.1684	0.2244
$CR_{20}$	0.3553	0.1087	0.2401	0.2536	0.0423
$CR_{21}$	0.2379	0.1210	0.2137	0.2137	0.2137
$CR_{22}$	0.1864	0.2125	0.2076	0.2070	0.1864

**Table 4:** Evaluation matrix by $DM_{2}$.

	$B_{1}$	$B_{2}$	$B_{3}$	$B_{4}$	$B_{5}$
$CR_{1}$	0.1369	0.2839	0.0821	0.2279	0.2691
$CR_{2}$	0.0892	0.2625	0.2475	0.2080	0.1928
$CR_{3}$	0.1818	0.2554	0.0779	0.2554	0.2294
$CR_{4}$	0.2677	0.3335	0.0375	0.0375	0.3237
$CR_{5}$	0.0405	0.4044	0.2254	0.2254	0.1042
$CR_{6}$	0.2055	0.1468	0.2055	0.1468	0.2956
$CR_{7}$	0.0716	0.2477	0.2348	0.2348	0.2109
$CR_{8}$	0.2718	0.0923	0.2718	0.2718	0.0923
$CR_{9}$	0.2024	0.2176	0.2052	0.1724	0.2024
$CR_{10}$	0.2421	0.2252	0.0320	0.3190	0.1817
$CR_{11}$	0.0978	0.1630	0.3207	0.0978	0.3207
$CR_{12}$	0.1804	0.1708	0.1288	0.2673	0.2527
$CR_{13}$	0.2041	0.1832	0.2187	0.1970	0.1970
$CR_{14}$	0.0380	0.2982	0.0380	0.3380	0.2878
$CR_{15}$	0.1258	0.1761	0.2303	0.2610	0.2067
$CR_{16}$	0.1064	0.1064	0.3678	0.1064	0.3132
$CR_{17}$	0.2013	0.2013	0.2365	0.1595	0.2013
$CR_{18}$	0.2329	0.1574	0.2336	0.2098	0.1663
$CR_{19}$	0.1401	0.1961	0.2821	0.1857	0.1961
$CR_{20}$	0.2704	0.0827	0.1828	0.1930	0.2711
$CR_{21}$	0.2323	0.1181	0.2087	0.2087	0.2323
$CR_{22}$	0.1864	0.2125	0.2076	0.2070	0.1864

**Table 5:** Evaluation matrix by $DM_{3}$.

	$B_{1}$	$B_{2}$	$B_{3}$	$B_{4}$	$B_{5}$
$CR_{1}$	0.1794	0.3723	0.1076	0.2988	0.0419
$CR_{2}$	0.0892	0.2625	0.2475	0.2080	0.1928
$CR_{3}$	0.1708	0.2589	0.0790	0.2589	0.2325
$CR_{4}$	0.2082	0.2593	0.2517	0.0292	0.2517
$CR_{5}$	0.0391	0.3905	0.2349	0.2349	0.1007
$CR_{6}$	0.2523	0.1801	0.1801	0.1286	0.2590
$CR_{7}$	0.0743	0.2570	0.2436	0.2063	0.2188
$CR_{8}$	0.2214	0.1024	0.2389	0.3348	0.1024
$CR_{9}$	0.0775	0.2736	0.2150	0.1807	0.2532
$CR_{10}$	0.2320	0.2576	0.0306	0.3057	0.1741
$CR_{11}$	0.2251	0.1487	0.2089	0.2084	0.2089
$CR_{12}$	0.1478	0.1510	0.2414	0.2364	0.2234
$CR_{13}$	0.2128	0.1910	0.2280	0.1628	0.2054
$CR_{14}$	0.0308	0.2418	0.2200	0.2740	0.2333
$CR_{15}$	0.1317	0.1844	0.2411	0.1844	0.2584
$CR_{16}$	0.2353	0.1865	0.2764	0.0799	0.2219
$CR_{17}$	0.1942	0.2470	0.2419	0.0699	0.2470
$CR_{18}$	0.2214	0.1994	0.2219	0.1994	0.1580
$CR_{19}$	0.1286	0.1801	0.2590	0.1801	0.2523
$CR_{20}$	0.2129	0.2258	0.1694	0.1789	0.2129
$CR_{21}$	0.2323	0.1181	0.2087	0.2087	0.2323
$CR_{22}$	0.1864	0.2125	0.2076	0.2070	0.1864

**Table 6:** The criteria weight after modified.

	$w_{CR,j}^{1}$	$w_{CR,j}^{2}$	$w_{CR,j}^{3}$
$CR_{1}$	0.0831	0.0822	0.0906
$CR_{2}$	0.0382	0.0382	0.0384
$CR_{3}$	0.0845	0.0823	0.0826
$CR_{4}$	0.0632	0.0626	0.0476
$CR_{5}$	0.0858	0.0879	0.0911
$CR_{6}$	0.0381	0.0381	0.0382
$CR_{7}$	0.0512	0.0509	0.0513
$CR_{8}$	0.0429	0.0440	0.0445
$CR_{9}$	0.0374	0.0374	0.0379
$CR_{10}$	0.0384	0.0377	0.0395
$CR_{11}$	0.0394	0.0365	0.0333
$CR_{12}$	0.0396	0.0395	0.0396
$CR_{13}$	0.0467	0.0467	0.0466
$CR_{14}$	0.0611	0.0615	0.0467
$CR_{15}$	0.0425	0.0425	0.0426
$CR_{16}$	0.0356	0.0391	0.0338
$CR_{17}$	0.0373	0.0373	0.0380
$CR_{18}$	0.0331	0.0332	0.0333
$CR_{19}$	0.0331	0.0331	0.0332
$CR_{20}$	0.0471	0.0399	0.0398
$CR_{21}$	0.0396	0.0396	0.0396
$CR_{22}$	0.0375	0.0375	0.0375

**Table 7:** The comprehensive weighted body of evidence given by $DM_{1}$.

${\underline{m}}_{j}^{1}\left(B_{i}\right)$	$B_{1}$	$B_{2}$	$B_{3}$	$B_{4}$	$B_{5}$
$CR_{1}$	0.1985	0.2008	0.2002	0.1999	0.2006
$CR_{2}$	0.2003	0.1995	0.2003	0.2000	0.1999
$CR_{3}$	0.2004	0.2020	0.1992	0.1970	0.2014
$CR_{4}$	0.2011	0.2013	0.1979	0.1979	0.2019
$CR_{5}$	0.1970	0.2033	0.2002	0.2002	0.1993
$CR_{6}$	0.2000	0.1996	0.2000	0.1996	0.2008
$CR_{7}$	0.1994	0.2003	0.2002	0.2002	0.1999
$CR_{8}$	0.2000	0.2002	0.2000	0.2000	0.2000
$CR_{9}$	0.1999	0.2000	0.1999	0.2003	0.2000
$CR_{10}$	0.2003	0.2005	0.1987	0.2008	0.1998
$CR_{11}$	0.2008	0.1997	0.1987	0.1997	0.2011
$CR_{12}$	0.2005	0.1998	0.1994	0.1998	0.2005
$CR_{13}$	0.2000	0.1998	0.2002	0.2000	0.2000
$CR_{14}$	0.1980	0.2018	0.1980	0.2006	0.2016
$CR_{15}$	0.1996	0.2001	0.2007	0.2001	0.1996
$CR_{16}$	0.1992	0.1992	0.2006	0.2001	0.2008
$CR_{17}$	0.2000	0.2000	0.1996	0.2002	0.2000
$CR_{18}$	0.2004	0.1998	0.1996	0.2003	0.1999
$CR_{19}$	0.1995	0.2002	0.2004	0.1998	0.2002
$CR_{20}$	0.2015	0.1991	0.2004	0.2005	0.1985
$CR_{21}$	0.2003	0.1994	0.2001	0.2001	0.2001
$CR_{22}$	0.1999	0.2001	0.2001	0.2001	0.1999

**Table 8:** The comprehensive weighted body of evidence given by $DM_{2}$.

${\underline{m}}_{j}^{2}\left(B_{i}\right)$	$B_{1}$	$B_{2}$	$B_{3}$	$B_{4}$	$B_{5}$
$CR_{1}$	0.1989	0.2015	0.1979	0.2005	0.2012
$CR_{2}$	0.1991	0.2005	0.2004	0.2001	0.1999
$CR_{3}$	0.1997	0.2010	0.1978	0.2010	0.2005
$CR_{4}$	0.2009	0.2018	0.1979	0.1979	0.2016
$CR_{5}$	0.1970	0.2039	0.2005	0.2005	0.1982
$CR_{6}$	0.2000	0.1996	0.2000	0.1996	0.2008
$CR_{7}$	0.1986	0.2005	0.2004	0.2004	0.2001
$CR_{8}$	0.2007	0.1990	0.2007	0.2007	0.1990
$CR_{9}$	0.2000	0.2001	0.2000	0.1998	0.2000
$CR_{10}$	0.2003	0.2002	0.1987	0.2009	0.1999
$CR_{11}$	0.1992	0.1997	0.2009	0.1992	0.2009
$CR_{12}$	0.1998	0.1998	0.1994	0.2005	0.2004
$CR_{13}$	0.2000	0.1998	0.2002	0.2000	0.2000
$CR_{14}$	0.1979	0.2013	0.1979	0.2018	0.2011
$CR_{15}$	0.1993	0.1998	0.2003	0.2005	0.2001
$CR_{16}$	0.1992	0.1992	0.2014	0.1992	0.2009
$CR_{17}$	0.2000	0.2000	0.2003	0.1997	0.2000
$CR_{18}$	0.2002	0.1997	0.2002	0.2001	0.1998
$CR_{19}$	0.1996	0.2000	0.2006	0.1999	0.2000
$CR_{20}$	0.2006	0.1990	0.1999	0.1999	0.2006
$CR_{21}$	0.2003	0.1993	0.2001	0.2001	0.2003
$CR_{22}$	0.1999	0.2001	0.2001	0.2001	0.1999

**Table 9:** The comprehensive weighted body of evidence given by $DM_{3}$.

${\underline{m}}_{j}^{3}\left(B_{i}\right)$	$B_{1}$	$B_{2}$	$B_{3}$	$B_{4}$	$B_{5}$
$CR_{1}$	0.1996	0.2034	0.1982	0.2019	0.1969
$CR_{2}$	0.1991	0.2005	0.2004	0.2001	0.1999
$CR_{3}$	0.1995	0.2010	0.1979	0.2010	0.2006
$CR_{4}$	0.2001	0.2006	0.2005	0.1983	0.2005
$CR_{5}$	0.1968	0.2037	0.2007	0.2007	0.1980
$CR_{6}$	0.2004	0.1998	0.1998	0.1994	0.2005
$CR_{7}$	0.1987	0.2006	0.2005	0.2001	0.2002
$CR_{8}$	0.2002	0.1991	0.2004	0.2012	0.1991
$CR_{9}$	0.1990	0.2006	0.2001	0.1998	0.2004
$CR_{10}$	0.2003	0.2005	0.1986	0.2009	0.1998
$CR_{11}$	0.2002	0.1996	0.2001	0.2001	0.2001
$CR_{12}$	0.1996	0.1996	0.2003	0.2003	0.2002
$CR_{13}$	0.2001	0.1999	0.2003	0.1996	0.2001
$CR_{14}$	0.1984	0.2004	0.2002	0.2007	0.2003
$CR_{15}$	0.1994	0.1999	0.2004	0.1999	0.2005
$CR_{16}$	0.2002	0.1999	0.2005	0.1992	0.2002
$CR_{17}$	0.2000	0.2004	0.2003	0.1990	0.2004
$CR_{18}$	0.2001	0.2000	0.2002	0.2000	0.1997
$CR_{19}$	0.1995	0.1999	0.2004	0.1999	0.2004
$CR_{20}$	0.2001	0.2002	0.1997	0.1998	0.2001
$CR_{21}$	0.2003	0.1993	0.2001	0.2001	0.2003
$CR_{22}$	0.1999	0.2001	0.2001	0.2001	0.1999

**Table 10:** The individual comprehensive evidence body of Eps.

	$B_{1}$	$B_{2}$	$B_{3}$	$B_{4}$	$B_{5}$
${\underline{m}}^{1}\left(B_{i}\right)$	0.1966	0.2065	0.1942	0.1970	0.2056
${\underline{m}}^{2}\left(B_{i}\right)$	0.1915	0.2058	0.1953	0.2022	0.2052
${\underline{m}}^{3}\left(B_{i}\right)$	0.1915	0.2091	0.1995	0.2020	0.1979

**Table 11:** The final weights of the 22 criteria.

Criteria	Weight	Subcriteria	Subcriteria Weight
Eco-environment	0.3909	$CR_{1}$	0.0853
		$CR_{2}$	0.0383
		$CR_{3}$	0.0831
		$CR_{4}$	0.0578
		$CR_{5}$	0.0883
		$CR_{6}$	0.0381
Tourist facilities	0.2937	$CR_{7}$	0.0511
		$CR_{8}$	0.0438
		$CR_{9}$	0.0376
		$CR_{10}$	0.0385
		$CR_{11}$	0.0364
		$CR_{12}$	0.0396
		$CR_{13}$	0.0466
Management level	0.2391	$CR_{14}$	0.0564
		$CR_{15}$	0.0425
		$CR_{16}$	0.0362
		$CR_{17}$	0.0375
		$CR_{18}$	0.0332
		$CR_{19}$	0.0331
Participant attitudes	0.1193	$CR_{20}$	0.0423
		$CR_{21}$	0.0396
		$CR_{22}$	0.0375

**Table 12:** The comprehensive decision matrix.

${\tilde{z}}_{ij}^{}$	$B_{1}$	$B_{2}$	$B_{3}$	$B_{4}$	$B_{5}$
$CR_{1}$	((3.0, 5.0, 7.0); 0.6, 0.3)	((6.3, 8.1, 8.9); 0.8, 0.1)	((3.0001, 5.0001, 6.6667); 0.6, 0.3)	((6.7, 7.6, 9.3); 0.6, 0.2)	((6.0, 7.0, 7.6667); 0.6, 0.3)
$CR_{2}$	((3.4334, 4.9667, 6.5); 0.6, 0.3)	((5.6666, 7.6666, 9.0); 0.6, 0.3)	((6.7, 7.6, 9.3); 0.6, 0.2)	((5.0, 7.0, 9.0); 0.6, 0.3)	((7.4, 8.5, 9.2); 0.4, 0.4)
$CR_{3}$	((5.8, 7.5, 9.0667); 0.4, 0.4)	((9.0, 10.0, 10.0); 0.6, 0.3)	((1.6667, 3.6667, 5.6667); 0.6, 0.3)	((5.9999, 6.9999, 7.6666); 0.6, 0.3)	((7.0, 9.0, 10.0); 0.6, 0.3)
$CR_{4}$	((6.7, 7.6, 9.3); 0.6, 0.2)	((6.5333, 8.4, 9.2667); 0.8, 0.1)	((1.9333, 3.2667, 5.0667); 0.8, 0.1)	((0.0, 1.0, 3.0); 0.6, 0.3)	((5.8, 7.8, 9.2); 0.8, 0.1)
$CR_{5}$	((0.0, 1.0, 3.0); 0.6, 0.3)	((8.3, 8.9, 9.5); 0.8, 0.1)	((6.6, 8.0, 9.1333); 0.6, 0.3)	((6.6, 8.0, 9.1333); 0.6, 0.3)	((1.6667, 3.6667, 5.6667); 0.6, 0.3)
$CR_{6}$	((6.0, 7.5, 9.0); 0.7, 0.2)	((3.6667, 5.6667, 7.6667); 0.6, 0.3)	((5.0, 7.0, 9.0); 0.6, 0.3)	((3.0, 5.0, 7.0); 0.6, 0.3)	((5.8, 7.8, 9.2); 0.8, 0.1)
$CR_{7}$	((3.1334, 4.8334, 6.4); 0.6, 0.3)	((6.3, 8.1, 8.9); 0.8, 0.1)	((9.0, 10.0, 10.0); 0.6, 0.3)	((8.2333, 9.2, 9.7667); 0.6, 0.2)	((7.0, 9.0, 10.0); 0.6, 0.3)
$CR_{8}$	((7.1333, 8.8333, 9.7333); 0.4, 0.4)	((3.6667, 5.3334, 6.6667); 0.6, 0.3)	((6.3333, 8.3333, 9.6667); 0.6, 0.3)	((7.3333, 8.8333, 9.6667); 0.7, 0.2)	((3.0001, 5.0001, 6.6667); 0.6, 0.3)
$CR_{9}$	((5.8666, 6.9333, 8.0); 0.6, 0.3)	((7.5, 9.0667, 9.9333); 0.7, 0.2)	((6.7, 7.6, 9.3); 0.6, 0.2)	((5.4333, 7.3667, 8.9667); 0.6, 0.3)	((7.7667, 8.8, 9.5); 0.7, 0.2)
$CR_{10}$	((7.0, 9.0, 10.0); 0.6, 0.3)	((8.1, 8.6333, 9.1667); 0.7, 0.2)	((0.0, 1.0, 3.0); 0.6, 0.3)	((8.3, 8.9, 9.5); 0.8, 0.1)	((6.0, 8.0, 9.0); 0.5, 0.4)
$CR_{11}$	((5.5001, 7.0667, 8.2667); 0.7, 0.2)	((3.6667, 5.6667, 7.6667); 0.6, 0.3)	((5.9999, 6.9999, 7.6666); 0.6, 0.3)	((4.0, 5.5, 7.0); 0.7, 0.2)	((9.0, 10.0, 10.0); 0.6, 0.3)
$CR_{12}$	((7.1334, 8.5, 9.4); 0.4, 0.4)	((6.0, 8.0, 9.0); 0.5, 0.4)	((4.8333, 6.4, 7.9333); 0.7, 0.2)	((6.2, 8.0667, 8.9333); 0.8, 0.1)	((8.0, 8.5, 9.0); 0.7, 0.2)
$CR_{13}$	((7.5, 8.5, 9.5); 0.6, 0.2)	((8.3, 8.9, 9.5); 0.8, 0.6)	((8.0, 8.5, 9.0); 0.7, 0.2)	((6.3333, 8.3333, 9.6667); 0.6, 0.3)	((7.0, 9.0, 10.0); 0.6, 0.3)
$CR_{14}$	((0.0, 1.0, 3.0); 0.6, 0.3)	((7.5, 8.5, 9.5); 0.6, 0.2)	((2.2333, 3.2, 5.1); 0.6, 0.2)	((6.2, 8.0667, 8.9333); 0.8, 0.1)	((7.0, 9.0, 10.0); 0.6, 0.3)
$CR_{15}$	((3.0, 5.0, 7.0); 0.6, 0.3)	((5.0, 7.0, 9.0); 0.6, 0.3)	((7.5, 8.5, 9.5); 0.6, 0.2)	((5.4333, 7.3667, 8.9667); 0.6, 0.3)	((6.4333, 7.4666, 8.5); 0.7, 0.2)
$CR_{16}$	((3.0, 5.0, 6.6667); 0.6, 0.3)	((2.3333, 4.3333, 6.3333); 0.6, 0.3)	((6.5333, 8.4, 9.2667); 0.8, 0.1)	((2.6667, 4.6667, 6.3334); 0.6, 0.3)	((7.2333, 8.3667, 9.4333); 0.6, 0.2)
$CR_{17}$	((6.9, 8.5333, 9.7667); 0.6, 0.2)	((7.5, 9.0667, 9.9333); 0.7, 0.2)	((6.2, 8.0667, 8.9333); 0.8, 0.1)	((5.0, 6.6667, 8.0); 0.6, 0.3)	((7.5, 9.0667, 9.9333); 0.7, 0.2)
$CR_{18}$	((8.0, 8.5, 9.0); 0.7, 0.2)	((6.3333, 8.3333, 9.3333); 0.6, 0.3)	((6.9999, 8.3333, 9.0); 0.6, 0.3)	((7.0, 9.0, 10.0); 0.6, 0.3)	((5.0, 7.0, 9.0); 0.6, 0.3)
$CR_{19}$	((3.0, 5.0, 7.0); 0.6, 0.3)	((5.6667, 7.6667, 9.3333); 0.6, 0.3)	((5.8, 7.8, 9.2); 0.8, 0.1)	((5.6667, 7.6667, 9.0); 0.6, 0.3)	((6.6667, 8.1667, 9.3333); 0.7, 0.2)
$CR_{20}$	((7.5667, 8.2, 9.1); 0.6, 0.2)	((3.0, 5.0, 6.6667); 0.6, 0.3)	((6.0, 8.0, 9.0); 0.5, 0.4)	((5.0, 7.0, 9.0); 0.6, 0.3)	((5.2332, 6.1999, 7.4333); 0.6, 0.2)
$CR_{21}$	((9.0, 10.0, 10.0); 0.6, 0.3)	((3.0, 5.0, 7.0); 0.6, 0.3)	((7.0, 9.0, 10.0); 0.6, 0.3)	((7.0, 9.0, 10.0); 0.6, 0.3)	((8.3333, 9.6667, 10.0); 0.6, 0.3)
$CR_{22}$	((7.0, 9.0, 10.0); 0.6, 0.3)	((5.8, 7.8, 9.2); 0.8, 0.1)	((9.0, 10.0, 10.0); 0.6, 0.3)	((8.0, 8.5, 9.0); 0.7, 0.2)	((7.0, 9.0, 10.0); 0.6, 0.3)

**Table 13:** The values $S\left(B_{i}\right)$, $R\left(B_{i}\right)$ and $Q\left(B_{i}\right)$ of five alternatives.

	$S\left(B_{i}\right)$	$R\left(B_{i}\right)$	$Q\left(B_{i}\right)$	Final Ranking
$B_{1}$	0.5701	0.9747	0.0128	1
$B_{2}$	0.3652	0.9600	0.6883	4
$B_{3}$	0.4734	0.9753	0.1765	2
$B_{4}$	0.4343	0.9720	0.3164	3
$B_{5}$	0.2961	0.9510	1.0000	5

**Table 14:** The ranking orders of alternatives with different θ.

θ	$Q\left(B_{1}\right)$	$Q\left(B_{2}\right)$	$Q\left(B_{3}\right)$	$Q\left(B_{4}\right)$	$Q\left(B_{5}\right)$	Ranking Orders	Best Candidates
0.1	0.0231	0.6405	0.0353	0.1732	1.0000	$B_{1}>B_{3}>B_{4}>B_{2}>B_{5}$	$B_{1}$
0.2	0.0205	0.6524	0.0706	0.2090	1.0000	$B_{1}>B_{3}>B_{4}>B_{2}>B_{5}$	$B_{1}$
0.3	0.0180	0.6644	0.1059	0.2448	1.0000	$B_{1}>B_{3}>B_{4}>B_{2}>B_{5}$	$B_{1}$
0.4	0.0154	0.6763	0.1412	0.2806	1.0000	$B_{1}>B_{3}>B_{4}>B_{2}>B_{5}$	$B_{1}$
0.5	0.0128	0.6883	0.1765	0.3164	1.0000	$B_{1}>B_{3}>B_{4}>B_{2}>B_{5}$	$B_{1}$
0.6	0.0103	0.7002	0.2118	0.3522	1.0000	$B_{1}>B_{3}>B_{4}>B_{2}>B_{5}$	$B_{1}$
0.7	0.0077	0.7122	0.2471	0.3881	1.0000	$B_{1}>B_{4}>B_{3}>B_{2}>B_{5}$	$B_{1}$
0.8	0.0051	0.7241	0.2824	0.4239	1.0000	$B_{1}>B_{4}>B_{3}>B_{2}>B_{5}$	$B_{1}$
0.9	0.0026	0.7361	0.3177	0.4597	1.0000	$B_{1}>B_{4}>B_{3}>B_{2}>B_{5}$	$B_{1}$

## Appendix A

### Appendix A.1. Proof Definition 3

Definition 3 can be proved by Mathematical induction

Assume that decision-maker weights are known

First, let n=2, according to $\gamma \tilde{a}=\left(\left(\gamma \underline{a},\gamma a,\gamma \bar{a}\right);\omega_{\tilde{a}},u_{\tilde{a}}\right)\;\left(\gamma >0\right)$

Where i=1,2.

Therefore, we can obtain,

$\begin{array}{l} TI-WAA\left({\tilde{a}}_{1},{\tilde{a}}_{2}\right)\\ =\left(\left(\gamma_{1}{\underline{a}}_{1}+\gamma_{2}{\underline{a}}_{2},\gamma_{1}a_{1}+\gamma_{2}a_{2},\gamma_{1}{\bar{a}}_{1}+\gamma_{2}{\bar{a}}_{2}\right);\underset{}{\min}\left\{\omega_{1},\omega_{2}\right\},\underset{}{\max}\left\{u_{1},u_{2}\right\}\right) \end{array}$

Then, let n=k

$\begin{array}{l} TI-WAA\left({\tilde{a}}_{1},{\tilde{a}}_{2},...,{\tilde{a}}_{k}\right)\\ =\left(\left(\gamma_{1}{\underline{a}}_{1}+\gamma_{2}{\underline{a}}_{2}+,...,+\gamma_{k}{\underline{a}}_{k},\gamma_{1}a_{1}+\gamma_{2}a_{2}+,...,+\gamma_{k}a_{k},\gamma_{1}{\bar{a}}_{1}+\gamma_{2}{\bar{a}}_{2},...,+\gamma_{k}{\bar{a}}_{k}\right);\underset{}{\min}\left\{\omega_{1},...,\omega_{k}\right\},\underset{}{\max}\left\{u_{1},...,u_{k}\right\}\right)\\ \left(\left(\sum_{r=1}^{k}w_{r}{\underline{a}}_{r},\sum_{r=1}^{k}w_{r}a_{r},\sum_{r=1}^{k}w_{r}{\bar{a}}_{r}\right);\underset{k}{\min}\left\{\omega_{1},\omega_{2},...,\omega_{k}\right\},\underset{k}{\max}\left\{\omega_{1},\omega_{2},...,\omega_{k}\right\}\right) \end{array}$

Finally, let n=k+1

$\begin{array}{l} TI-WAA\left({\tilde{a}}_{1},{\tilde{a}}_{2},...,{\tilde{a}}_{k},{\tilde{a}}_{k+1}\right)\\ =\left(\left(\gamma_{1}{\underline{a}}_{1}+\gamma_{2}{\underline{a}}_{2}+,...,+\gamma_{k+1}{\underline{a}}_{k+1},\gamma_{1}a_{1}+\gamma_{2}a_{2}+,...,+\gamma_{k+1}a_{k+1},\gamma_{1}{\bar{a}}_{1}+\gamma_{2}{\bar{a}}_{2},...,+\gamma_{k+1}{\bar{a}}_{k+1}\right);\underset{}{\min}\left\{\omega_{1},...,\omega_{k+1}\right\},\underset{}{\max}\left\{u_{1},...,u_{k+1}\right\}\right)\\ =\left(\left(\sum_{r=1}^{k+1}w_{r}{\underline{a}}_{r},\sum_{r=1}^{k+1}w_{r}a_{r},\sum_{r=1}^{k+1}w_{r}{\bar{a}}_{r}\right);\underset{k+1}{\min}\left\{\omega_{1},\omega_{2},...,\omega_{k},\omega_{k+1}\right\},\underset{k+1}{\max}\left\{\omega_{1},\omega_{2},...,\omega_{k},\omega_{k+1}\right\}\right) \end{array}$

### Appendix A.2. Proof Property (3)

$\{\begin{array}{c} \left|\omega_{1}-\omega_{2}\right|+\left|\omega_{2}-\omega_{3}\right|\geq \left|\omega_{1}-\omega_{\tilde{b}}\right|\\ \left|u_{1}-u_{2}\right|+\left|u_{2}-u_{3}\right|\geq \left|u_{1}-u_{\tilde{b}}\right| \end{array}$

Then

$\max\left\{\left|\omega_{1}-\omega_{2}\right|,\left|u_{1}-u_{2}\right|\right\}+\max\left\{\left|\omega_{2}-\omega_{3}\right|,\left|\omega_{1}-\omega_{\tilde{b}}\right|\right\}\geq \left\{\left|u_{2}-u_{3}\right|,\left|u_{1}-u_{\tilde{b}}\right|\right\}$

And because

$\{\begin{array}{c} \left|{\underline{a}}_{1}-{\underline{a}}_{2}\right|+\left|{\underline{a}}_{2}-\underline{b}\right|\geq \left|{\underline{a}}_{1}-\underline{b}\right|\\ \left|a_{1}-a_{2}\right|+\left|a_{2}-b\right|\geq \left|a_{1}-b\right|\\ \left|{\bar{a}}_{1}-{\bar{a}}_{2}\right|+\left|{\bar{a}}_{2}-\bar{b}\right|\geq \left|{\bar{a}}_{1}-\bar{b}\right| \end{array}$

Hence $d_{h}\left({\tilde{a}}_{1},\tilde{b}\right)\leq d_{h}\left({\tilde{a}}_{1},\tilde{b}\right)+d_{h}\left({\tilde{a}}_{2},\tilde{b}\right)$ Similarly, $d_{e}\left({\tilde{a}}_{1},\tilde{b}\right)\leq d_{e}\left({\tilde{a}}_{1},\tilde{b}\right)+d_{e}\left({\tilde{a}}_{2},\tilde{b}\right)$ can also be proved.
